# From hair to liver: emerging application of hair follicle mesenchymal stem cell transplantation reverses liver cirrhosis by blocking the TGF-β/Smad signaling pathway to inhibit pathological HSC activation

**DOI:** 10.7717/peerj.12872

**Published:** 2022-02-15

**Authors:** Qi Liu, Chengqian Lv, Yanan Jiang, Kunpeng Luo, Yang Gao, Jingyang Liu, Xu Zhang, Jan Mohammad Omar, Shizhu Jin

**Affiliations:** 1 Department of Gastroenterology and Hepatology, Second Affiliated Hospital of Harbin Medical University, Harbin, China; 2 Department of Pharmacology (State-Province Key Laboratories of Biomedicine-Pharmaceutics of China, Key Laboratory of Cardiovascular Research, Ministry of Education), College of Pharmacy of Harbin Medical University, Harbin, China; 3 Translational Medicine Research and Cooperation Center of Northern China, Heilongjiang Academy of Medical Sciences, Harbin, China

**Keywords:** Liver cirrhosis, Hair follicle mesenchymal stem cells, Homing, Differentiation, Hepatic stellate cells, TGF-β/Smad signaling pathway

## Abstract

Liver cirrhosis (LC) involves multiple systems throughout the body, and patients with LC often die of multiple organ failure. However, few drugs are useful to treat LC. Hair follicle mesenchymal stem cells (HF-MSCs) are derived from the dermal papilla and the bulge area of hair follicles and are pluripotent stem cells in the mesoderm with broad prospects in regenerative medicine. As an emerging seed cell type widely used in skin wound healing and plastic surgery, HF-MSCs show considerable prospects in the treatment of LC due to their proliferation and multidirectional differentiation capabilities. We established an LC model in C57BL/6J mice by administering carbon tetrachloride (CCl_4_) and injected HF-MSCs through the tail vein to explore the therapeutic effects and potential mechanisms of HF-MSCs on LC. Here, we found that HF-MSCs improved liver function and ameliorated the liver pathology of LC. Notably, PKH67-labeled HF-MSCs were detected in the injured liver and expressed the hepatocyte-specific markers cytokeratin 18 (CK18) and albumin (ALB). In addition, in contrast to that in the LC group, the α-SMA expression showed a decreasing trend in the treatment group *in vitro* and *in vivo*, indicating that the pathological activation of hepatic stellate cells (HSCs) was inhibited by HF-MSC treatment. Moreover, the levels of transforming growth factor β (TGF-β1) and p-Smad3, a signaling molecule downstream of TGF-β1, were increased in mice with LC, while HF-MSC treatment reversed these changes *in vivo* and *in vitro*. Based on these findings, HF-MSCs may reverse LC by blocking the TGF-β/Smad pathway and inhibiting the pathological activation of HSCs, which may provide evidence for the application of HF-MSCs to treat LC.

## Introduction

Liver cirrhosis (LC) is an advanced liver disease with a low survival rate and poor prognosis in the clinic. It is triggered by the continuous injury of pathogen infection, alcohol, drug, and chemical poisons (Goodman, 2007; Huang, Hu & Yang, 2010; Kisseleva, Gigante & Brenner, 2010; Silveira et al., 2010). More than 1 million people die from LC annually, and this disease has become a burden to public health (Rybicka et al., 2020). Liver transplantation is considered the best choice for the treatment of LC by removing the diseased liver that has lost its function and implanting healthy liver tissue into the recipients, but its application is limited due to a series of operation-related complications, shortage of donors, anti-host reaction, and high medical cost (Linecker et al., 2018; Zhang et al., 2020). Therefore, the identification of a safe and effective alternative method that can be widely used in LC treatment is urgently needed.

Recent data have indicated that termination of the fibrotic process reverses advanced fibrosis and even cirrhosis (Jung & Yim, 2017). The pathological activation of hepatic stellate cells (HSCs) is closely related to cirrhosis, which is considered the most important process in the development of LC (Friedman, 2008; Ma et al., 2016). During LC development, HSCs are pathologically activated and are induced to transform into contractile, proliferative and fibroblastic myofibroblasts, leading to the accumulation of collagen and other extracellular matrix components (Friedman, 2000). Pathological HSC activation is regulated by a variety of cytokines (Lee et al., 2004; Li, Wang & Chen, 2017), among which transforming growth factor β (TGF-β) is one of the most effective cytokines inducing fibrogenesis (Hellerbrand et al., 1999). Based on the description above, blocking the TGF-β1-activated Smad signaling pathway represents a potential therapeutic target for regulating HSC pathological activation and reversing LC (Dooley et al., 2003; Schnabl et al., 2001).

Mesenchymal stem cells (MSCs) show considerable clinical application value in tissue repair. MSCs are nonterminally differentiated cells with the functions of self-proliferation, multilineage differentiation, immune regulation, hematopoiesis induction, and the repair of damaged organs (Alfaifi et al., 2018; Srijaya, Ramasamy & Kasim, 2014) and have been shown to be effective in the treatment of liver fibrosis (Chiabotto et al., 2020). Bone marrow mesenchymal stem cells (BM-MSCs) have been the most extensively studied cells in cell therapy for liver fibrosis at present, but BM-MSCs have some limitations, such as the potential to cause damage to donors and small available quantities. Therefore, the identification of new sources of MSCs has become a hot topic in recent years.

The hair follicle is the basic unit of hair. A reservoir of hair follicle mesenchymal stem cells (HF-MSCs) is located in the bulge region of each hair follicle (Cotsarelis, 2006). Hair follicles are abundant in number, easy to obtain, do very little damage to the body and have low immunogenicity (Li et al., 2015). More importantly, they have a higher proliferative capacity than BM-MSCs (Bajpai, Mistriotis & Andreadis, 2012). In addition, as a type of MSC, HF-MSCs have the characteristics of differentiation and self-renewal *in vitro*. They potentially construct functional vascular grafts (Peng et al., 2011), hematopoietic tissues (Lako et al., 2002), and full-thickness skin (Han et al., 2007), deliver controlled-release insulin genes (Wu et al., 2015), repair peripheral nerve damage (Mii et al., 2013), and differentiate into hepatocyte-like cells (HLCs) (Shi et al., 2016; Xu et al., 2018), indicating that HF-MSCs have broad prospects in hepatic diseases. However, HF-MSCs have rarely been studied as promising cells in liver diseases.

We intended to investigate the therapeutic effects of HF-MSCs on LC and the potential mechanisms to provide theoretical support for the application of HF-MSCs in LC treatment.

## Materials & Methods

### Experimental animals

Wild-type healthy C57BL/6J mice (male, 20–22 g) were purchased from the Animal Center of the Second Affiliated Hospital of Harbin Medical University (Harbin, China). The mice were housed in the animal center for 1 week at 25 °C to adapt to the environment before the experiment. The mice were allowed to obtain feed and tap water normally. Our experimental protocol was performed according to medical ethics principles, and the mouse experiments were approved by the ethics committee of the Experimental Center of the Second Affiliated Hospital of Harbin Medical University (No. SYDW2019-240).

Humane endpoints were defined when mice showed persistent self-harm behavior and nonhealing wounds, and we euthanized the mice before the end of the experiment. Pentobarbital sodium was used to alleviate pain or fear during euthanasia, after which the mice were transferred to a new cage and placed in a quiet and undisturbed place. No animals survived at the end of the experiment.

### Isolation, culture, characterization and staining of HF-MSCs

The extraction of HF-MSCs was as reported previously (Sun et al., 2021). Six- to seven-day-old mice were used for the extraction of primary HF-MSCs. The mouse vibrissa pads were dissected and cut vertically into several small tissues. The tissues were then incubated with 0.1% type I collagenase (Sigma-Aldrich, St. Louis, MO, USA) at 37 °C for 4 h. The subcutaneous fat and connective tissue around the vibrissa were peeled off and then the hair follicles were separated from the connective tissue sheath with micro tweezers under a binocular microscope. The hair was cut above the bulge area. One or two hair follicles were inoculated in the middle of each well of a 24-well plate with complete medium, containing 15% fetal bovine serum (FBS) (ScienCell, San Diego, California, USA), 100 U/mL penicillin–streptomycin (Beyotime, Shanghai, China), and DMEM/F12 (Gibco, Gaithersburg, MD, USA), at 37 °C with 5% CO_2_. The cells were passaged when they reached 70–80% confluence, and the medium was changed the day before passaging to maintain cell viability. The operations described above involving cell culture were strictly performed under aseptic conditions.

We identified HF-MSCs by detecting the expression of the surface marker (CD29, CD90, CD31 and CD34) and their potential to differentiate into adipocytes and osteocytes. Third-passage HF-MSCs with good growth conditions were stained with the low-toxicity cell membrane dye PKH67 (Sigma–Aldrich) to locate HF-MSCs in the damaged liver after transplantation. Then, the stained HF-MSCs and their distribution in multiple organs were captured under an upright fluorescence microscope (BX51; Olympus, Japan).

### Animal model of cirrhosis and experimental design

**Figure 1:**
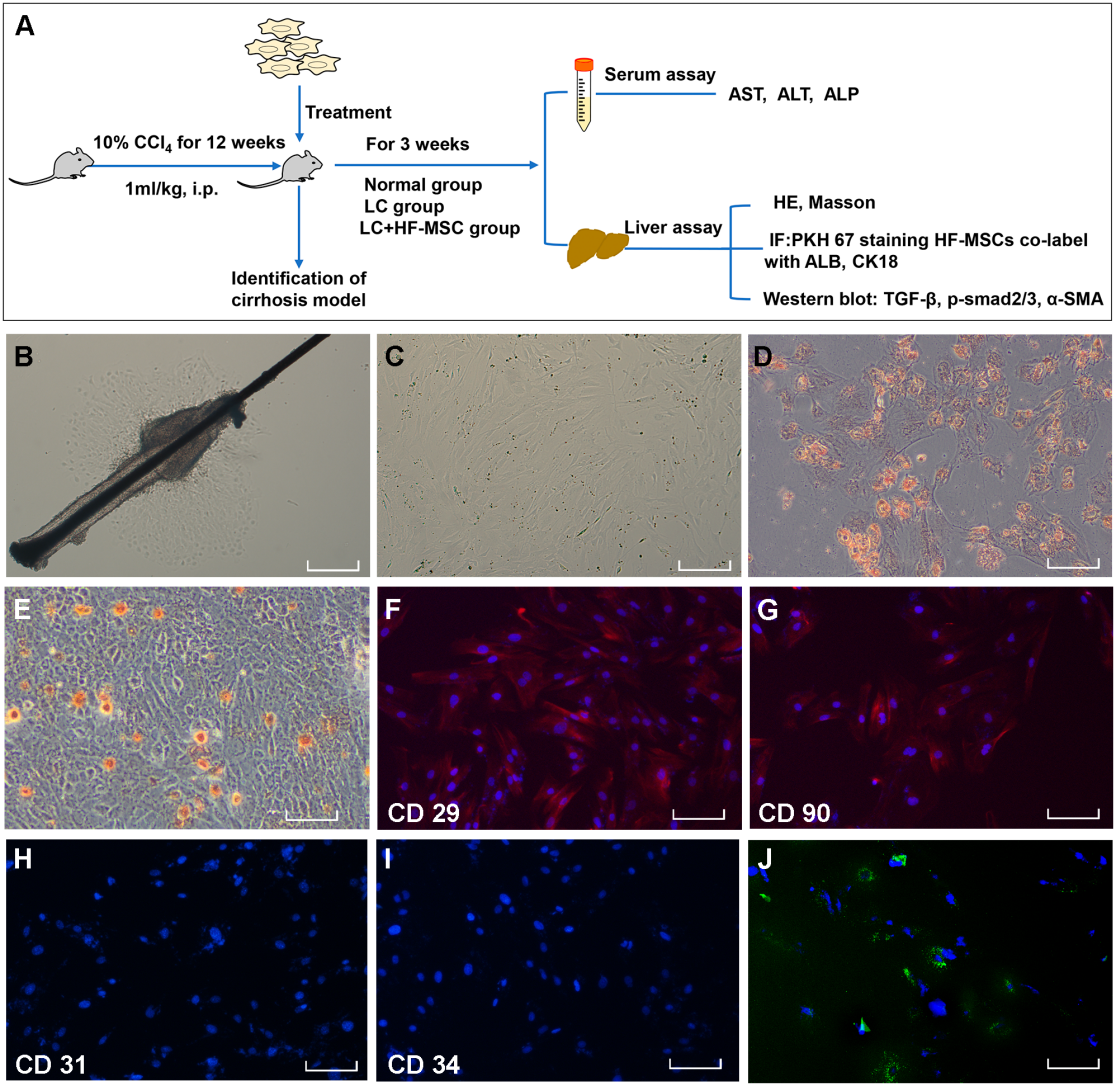
Extraction, identification and staining of HF-MSCs. (A) The experimental design; (B) Primary HF-MSCs migrated from the bulge area; (C) Second-generation HF-MSCs; (D, E) Adipogenic and osteogenic differentiation of HF-MSCs; (F–I) The extracted cells expressed CD29, CD90, while the expression of CD31 and CD34 was negative; (J) PKH67-labeled HF-MSCs show green light under a fluorescence microscope, and the nucleus is stained blue by DAPI. Scale bar (B–J) 50 µm.

The results of the preliminary experiment showed a significant difference when the sample size of mice was six. Thirty-five experimental mice were used in this study. carbon tetrachloride (CCl_4_) was intraperitoneally injected at a concentration of 10% to establish a mouse LC model with a dose of 1 mL/kg and a frequency of two times per week. Cirrhosis was evaluated by serology and histology after the last injection in the 12th week. Five mice that died during modeling were excluded from the experiment, and the surviving mice were used for subsequent experiments and analyses. Then, the CCl_4_-induced model mice were divided into the following two groups at random (based on a random order generator): (1) the HF-MSC group (*n* = 6), which was injected with 1 × 10^6^ HF-MSCs dissolved in PBS, and (2) the model group (*n* = 6), which was injected with an equivalent volume of saline. Mice in the control group (*n* = 6) were injected with 0.9% saline. Different groups of mice were assigned to different cages. Then the mice were administered 250 µl of the same injection volume by tail vein injection. Three weeks after cell transplantation, the liver tissues and serum obtained from the left ventricle were collected for subsequent analysis (Fig. 1A).

### Hepatic stellate cell line and TGF-β1-induced HSC activation

The immortalized murine hepatic stellate cell line JS1 (Otwo Biotech, ShenZhen, China) was used to explore the effect of HF-MSCs on HSCs *in vivo*. JS1 cells were activated by cocultured with TGF-β1 (Peprotech, NJ, USA) at a concentration of five ng/mL for 48 h (Ma et al., 2018). JS1 cells were cultured in complete medium consisting of high-glucose DMEM (Gibco). Then, the activated JS1 cells were used for the Transwell assay.

### Transwell assay

Transwell assays were performed to coculture JS1 cells and HF-MSCs in a double-cell coculture system using Transwell chambers with a 0.4 µm semipermeable membrane. HF-MSCs (2 × 10^5^ cells/well) were inoculated in the upper chamber of 6-well culture plates, and the same number of activated JS1 cells was mixed and inoculated in the lower chamber of the same well. HF-MSCs and IS1 cells were cocultured with high-glucose DMEM. JS1 cells alone were used as a control. After coculture for 48 h with saturated humidity and 5% CO_2_, cells in the lower chamber were harvested for a Western blot assay.

### Western blot analysis

Liver tissues from all groups that had been stored in liquid nitrogen were ground into a powder in a mortar and placed in an EP tube. Protease inhibitors and phosphatase inhibitors were added to lysis buffer to prevent the decomposition and dephosphorylation of the target proteins, and the entire lysis process was performed on ice for 30 min. The supernatant was transferred to an EP tube after centrifugation. After measuring the total protein concentration, the same amount of protein from each group was separated on 8% or 10% SDS–PAGE gels according to their molecular weights. Then, the proteins in each lane were transferred onto polyvinylidene difluoride membranes (EMD Millipore Corporation, Billerica, MA, USA) using a membrane transfer apparatus (Bio-Rad, Hercules, CA, USA). Blocking buffer (Bio-Rad) was used to block nonspecific binding. After an incubation with primary antibodies overnight, the membranes containing the proteins were then incubated with secondary antibodies for 1 h at room temperature. The following primary antibodies were used: anti-β-actin (ab8226, 1:1000; Abcam, Cambridge, UK), anti-TGF-β1 (#3711, 1:1000; Cell Signaling Technology, Beverly, MA, USA), anti-p-Smad2/3 (#8828, 1:1000, Cell Signaling Technology), anti-Smad2/3 (AF6367, 1:1500; Affinity, Changzhou, China) and anti-α-SMA (14395–1-AP, 1:2000; Proteintech, Wuhan, China). Images of the specific protein bands were captured using an Omega-Lum G imaging system after an incubation with enhanced chemiluminescence solution and analyzed using ImageJ software.

### Labeling HF-MSCs with DiR and *in vivo* imaging

DiR is a lipophilic fluorescent dye that can be used to stain cell membranes. DiR (D12731; Invitrogen) labeled HF-MSCs were used to track HF-MSCs *in vivo*. Adjust the third-generation HF-MSCs concentration to 1 × 10^6^/L, and add 5 uL of three mmol/L DIR storage solution to one mL of cell suspension. The DiR solution and cells were incubated for 30 min. Then the stained cells were transplanted via tail vein. After 48 h, the biodistribution of DiR-labeled HF-MSCs were performed by *in vivo* imaging System (Night OWL II LB983).

### Immunofluorescence staining

The frozen liver, kidney, lung and spleen tissues were sectioned to a thickness of 5–6 µm. Goat serum (abs933, Absin, Shanghai, China) was used to block the sections. The slices were incubated at 4 °C overnight with the following antibodies: anti-cytokeratin 18 (CK18) (ab181597, Abcam) and anti-albumin (ALB) (ab207327; Abcam). Sections were then incubated with a goat anti-rabbit secondary antibody (SA00013–4; Proteintech) for 1 h at room temperature on the second day. Next, after 3 rinses with TBST, the sections were incubated with 4,6-diamidino-2-phenylindole (DAPI; Beyotime) for 4 min. Then, the stained sections were mounted with antifade mounting medium.

### Pathological analysis

The dried paraffin-embedded liver tissue sections were completely immersed in xylene and different concentrations of ethanol and then rinsed several times with PBS. Slices were stained with an aqueous hematoxylin solution and differentiated with an acid solution in alcohol. After an incubation with ammonia, the slices were stained with eosin and dehydrated and sealed with neutral gum.

For Masson’s trichrome staining, the slices were stained with Weigert iron hematoxylin solution for 7 min. The sections were differentiated with an acidic ethanol differentiation solution for 6 s and then returned to blue with Masson’s blue solution for 4 min. After rinses with distilled water, the sections were stained with Ponceau red magenta solution for 5 min. The slices were washed with phosphomolybdic acid solution and then immersed in aniline blue staining solution for 2 min. After sealing with neutral gum, they were observed under a microscope.

### Serological analysis

Blood was collected from the left ventricle into a coagulation-accelerating vacuum tube when the mice were sacrificed. During blood collection, contamination with mouse hair was avoided, and the blood was directly collected into the bottom of the tubes to avoid hemolysis. The supernatant was collected after the blood coagulated in an EP tube. ELISA kits were used to evaluate serum ALB (ab207620; Abcam), alkaline phosphatase (ALP) (SEB472Mu; Cloud-Clone Corp, Wuhan, China), alanine aminotransferase (ALT) (CSB-E16539 m; Cusabio, Wuhan, China) and aspartate aminotransferase (AST) levels (ab263882; Abcam).

### Statistical analyses

At least three independent replicates were performed of each experiment. All data are presented as the means ± standard deviations (SD) and were analyzed using GraphPad Prism 8.0 software (GraphPad Prism Software, San Diego, CA, USA). Student’s t test was used for comparisons between two groups, and one-way ANOVA with Tukey’s test were used to determine significant differences between multiple groups. A nonparametric test was used for data that did not exhibit a normal distribution. Differences were considered statistically significant when *P* values were less than 0.05.

## Results

### Characteristics of HF-MSCs

The primary HF-MSCs showed adherent growth and a paving stone-like appearance on the fifth day of cell culture (Fig. 1B). Primary cells grew rapidly after adherence and were subcultured after 10 days. Cells of the second generation showed a typical spindle-shaped morphology (Fig. 1C). The multilineage differentiation potential of HF-MSCs was determined by performing osteogenesis and adipogenesis experiments (Figs. 1D–1E). The extracted cells expressed CD29, CD90, while the expression of CD31 and CD34 was negative (Figs. 1F–1I). Cells labeled with the PKH67 green fluorescent dye are shown in Fig. 1J. This result indicates that we successfully extracted HF-MSCs.

### Evaluation of the cirrhosis model

**Figure 2:**
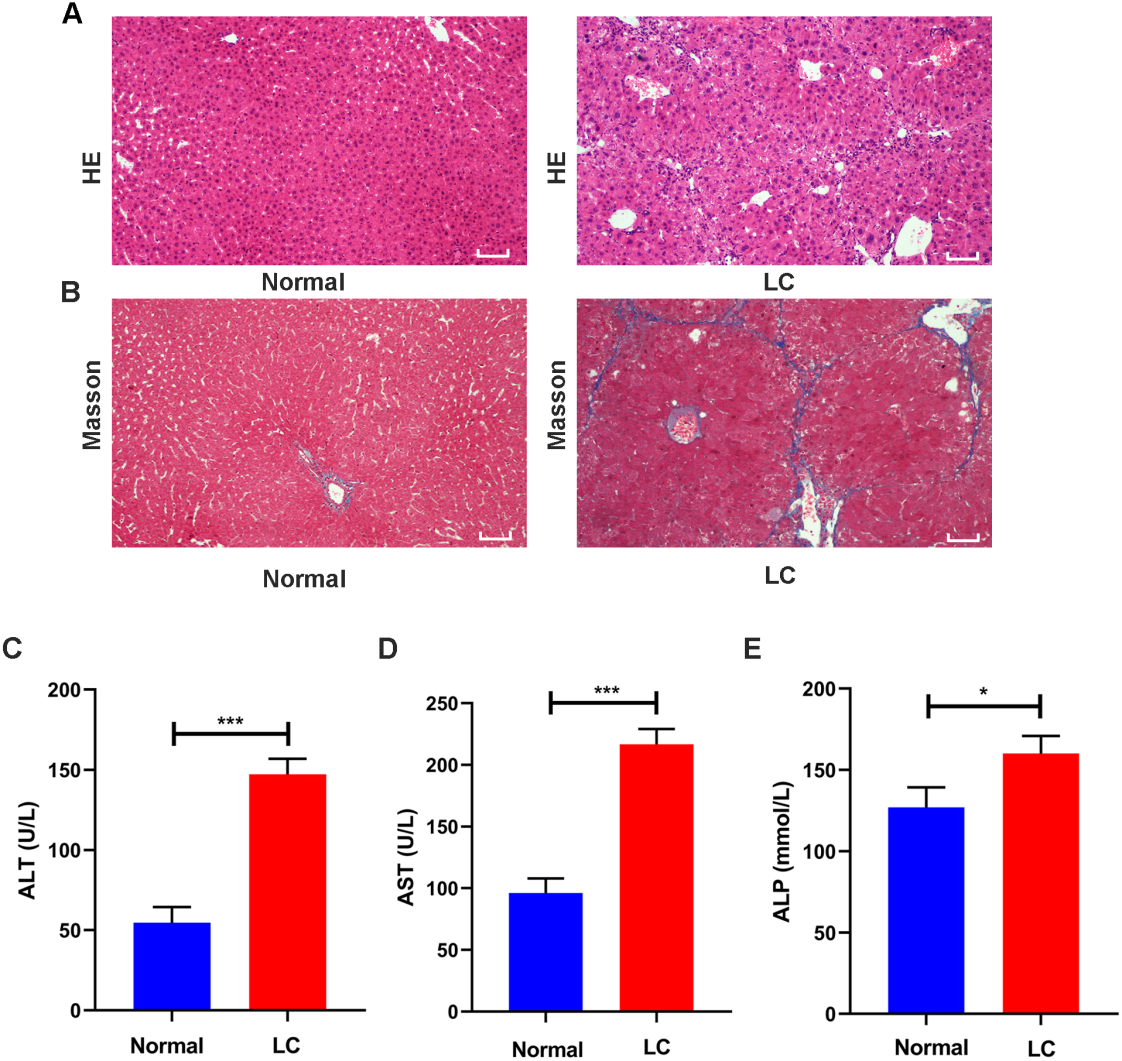
Validation of liver cirrhosis model. (A, B) HE and Masson staining between the normal group and the LC group (*n* = 6); (C–E) Changes in the levels of the serological indicators ALT, AST, and ALP in the normal and LC groups (*n* = 6); Scale bar (A, B) 50 µm. All data are shown as the means ± SD (Student’s *t*, *n* = 3). *P < 0.05, **P < 0.01, ***P < 0.001.

After 12 weeks of CCl_4_ administration, healthy mice and model mice were used to identify whether the model was successfully established. HE staining showed that the structure of hepatocytes in the normal group was clear without necrosis or inflammatory cell infiltration, and collagen fibers were rarely observed in the perisinusoidal space. In the model group, the arrangement of hepatocytes was altered, and the hepatocytes showed fatty degeneration and necrosis. Collagen fibers around the central vein in the liver tissue proliferated and formed pseudolobules (Figs. 2A–2B).

Serum assays were performed to assess hepatic function. The ALT, AST and ALP levels in the model group were increased to varying degrees compared with those in the normal group (all *P* values <0.05, Figs. 2C–2E). These features were consistent with the typical histopathological and serological changes of LC induced by CCl_4_.

### HF-MSCs alleviated histological injury and improved liver function in mice

**Figure 3:**
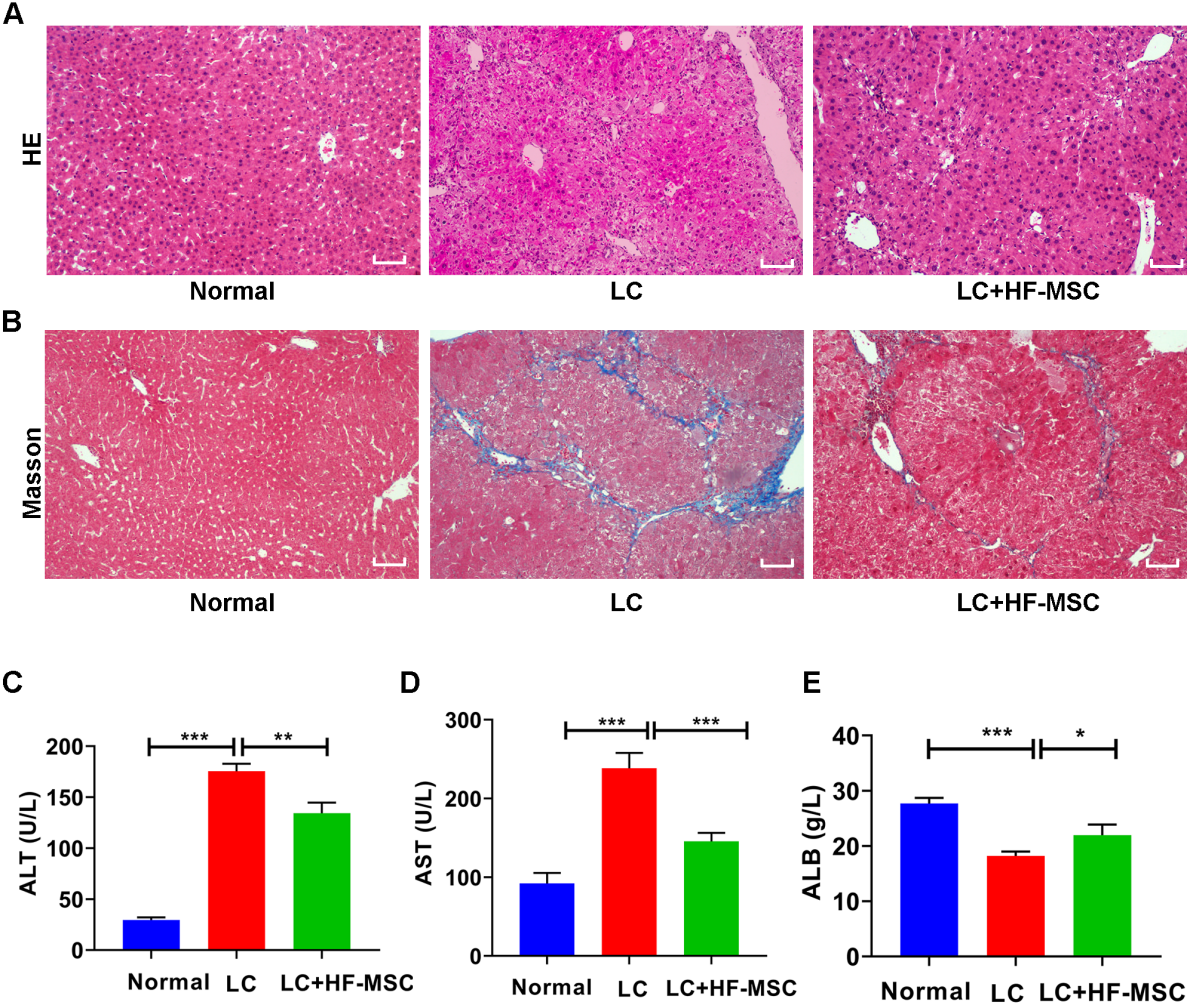
HF-MSCs relieved liver pathological damage and improved liver function. (A, B) HE staining and Masson staining in the three groups (*n* = 6). (C–E) Comparison of liver serological indices ALT, AST, and ALB in the three groups (*n* = 6). Scale bar (A, B) 50 µm. All data are shown as the means ± SD (One-way ANOVA, *n* = 3). *P < 0.05, **P < 0.01, ***P < 0.001.

The mice were sacrificed to collect liver tissue and serum after 3 weeks of cell therapy. The degree of LC was evaluated by performing HE and Masson’s trichrome staining. HE staining of hepatic paraffin sections from the model group showed degeneration and necrosis of hepatocytes and a large area of pseudolobular tissue structure, and Masson’s trichrome staining showed thick and deeply stained deposited collagen fibers extending outward from the portal area. However, these pathological changes related to LC were significantly attenuated by HF-MSC treatment (Figs. 3A–3B). Measurements of liver function indicated remarkable increases in the levels of ALT (175.49 ± 7.29 U/L) and AST (238.42 ± 19.44 U/L), while the serum level of ALB (18.22 ± 0.78 g/L) exhibited a decreasing trend in LC mice. However, these changes were reversed in the treatment group (Figs. 3C–3E). In summary, HF-MSCs promoted the repair of liver injury.

### Labeled HF-MSCs migrated to the injured liver and expressed hepatocyte-specific markers

**Figure 4:**
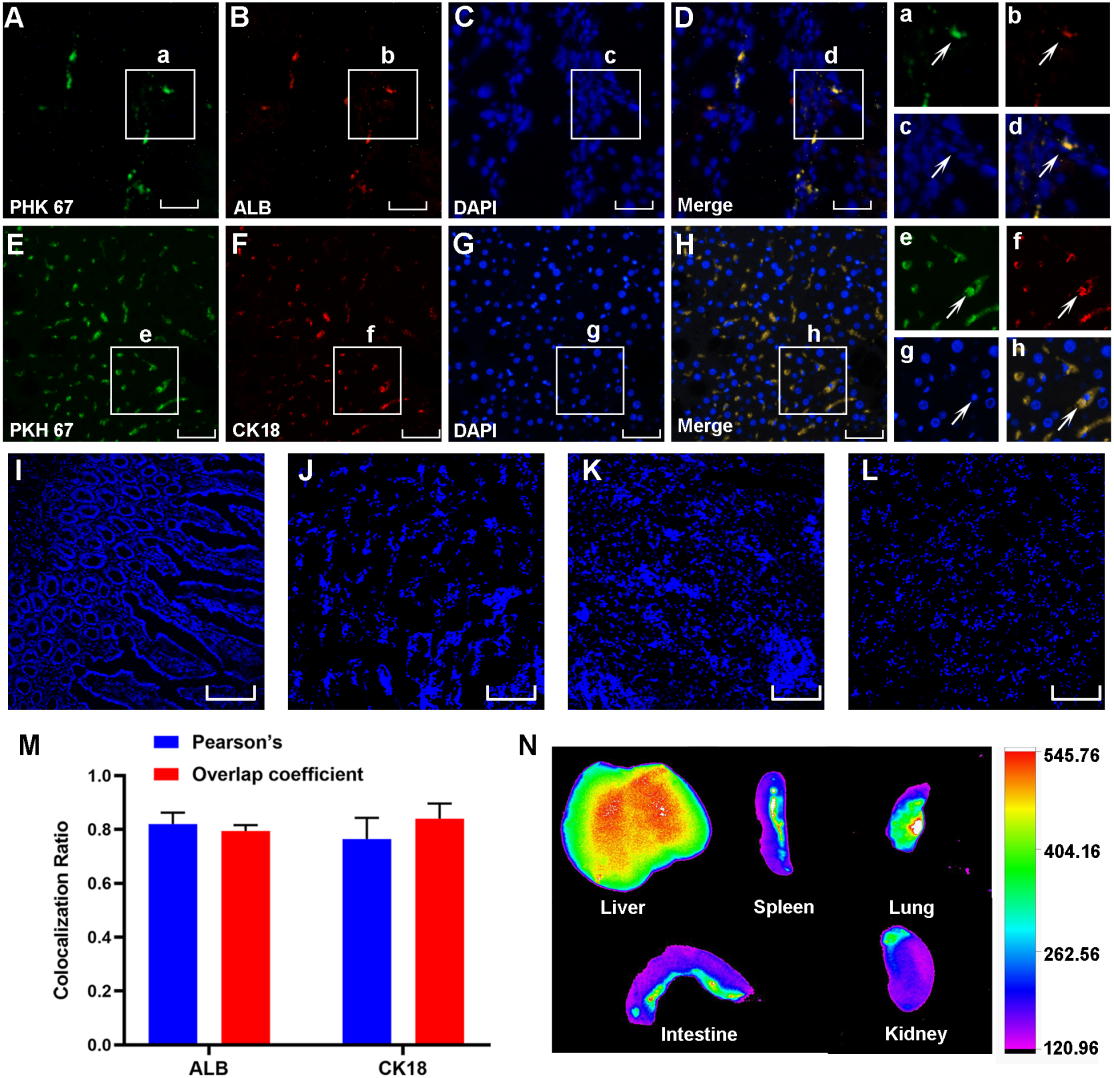
PKH67-labeled HF-MSCs homed to the damaged liver and expressed hepatocyte-specific surface markers. (A, E) PKH67-labeled HF-MSCs existed in the injured liver and are shown in green (*n* = 6). (B, F) Hepatocytes expressing the surface markers ALB and CK18 are shown in red (*n* = 6). (C, D, G, H) PKH67-stained HF-MSCs were colocalized with the hepatocyte-specific surface markers ALB and CK18 (*n* = 6). a–h show partially enlarged views of A–H. (I–L) PKH67-labeled HF-MSCs were rarely seen in the intestine, lung, spleen and kidney (*n* = 6). (M) Pearson’s correlation and the overlap coefficient of liver sections costained with ALB and CK18. (N) After transplanting the DiR-labeled HF-MSCs for 48 h, the HF-MSCs were mostly gathered in the lung and the liver. Scale bar: 50 µm (A–H), 200 µm (I–L).

Three weeks after cell transplantation, PKH67-labeled HF-MSCs resided in the injured liver tissue and showed green fluorescence (Figs. 4A–4E). The hepatocyte-specific markers CK18 and ALB were detected in liver sections from cirrhotic animals (Figs. 4B and 4F). More importantly, these labeled cells were exactly colocalized with the hepatocyte surface markers ALB and CK18 (Figs. 4D and 4H), indicating that HF-MSCs in the damaged liver expressed hepatocyte surface markers. Notably, PKH67-stained HF-MSCs were rarely observed in the intestine, kidney, lung and spleen tissues (Figs. 4I–4L). Pearson’s correlation coefficient and the overlap coefficient reflecting the degree of colocalization are shown in Fig. 4M. To detect the distribution of DiR-labeled HF-MSCs *in vivo*, the *in vivo* imaging was performed. It showed that after transplanting the DiR-labeled HF-MSCs for 48 h, the HF-MSCs were mostly gathered in the lung and the liver (Fig. 4N).

### HF-MSCs suppressed the pathological activation of HSCs and inhibited the TGF-β/Smad signaling pathway *in vitro*

**Figure 5:**
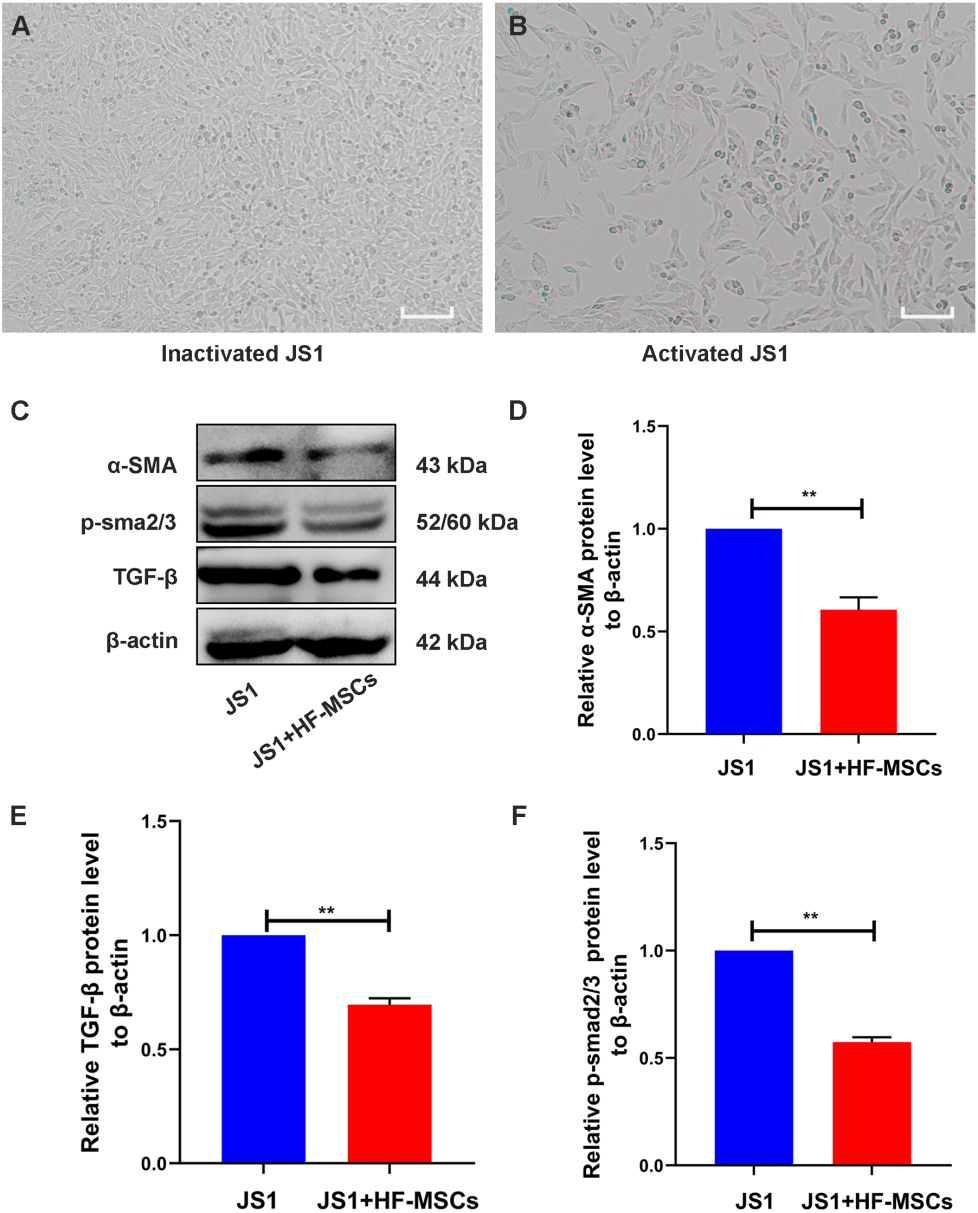
HF-MSCs inhibited HSC activation by inhibiting the TGF-β/Smad pathway *in vitro*. (A) Quiescent JS1 cells are in a static state, with irregular shapes, and the cell bodies are ovular or irregular; (B) The activated JS1 cells have enlarged cell bodies, protruding slender pseudopods, with a star-shaped appearance; (C) Immunoblotting analysis of α-SMA, TGF-β, p-Smad2/3 in the JS1 group and the JS1+HF-MSCs group (*n* = 6); (D) Semiquantitative analysis of α-SMA, TGF-β, p-Smad2/3. Scale bar (A, B) 50 µm. All data are shown as the means ± SD (One-way ANOVA, *n* = 3). *P < 0.05, **P < 0.01, ***P < 0.001.

The cell bodies of quiescent JS1 cells were oval or irregular (Fig. 5A). After HF-MSCs were cocultured with JS1 cells for 48 h, the activated JS1 cells displayed enlarged cell bodies, protruding slender pseudopods, and a star-shaped appearance (Fig. 5B). Compared with the JS1 group, the expression of α-SMA, an indicator of JS1 cell activation, was decreased in the cell treatment group (*P* < 0.05). Furthermore, levels of TGF-β1 and p-Smad3 in the JS1 group were noticeably increased, which were inhibited by cell treatment in the HF-MSC group (*P* < 0.05) (Figs. 5C–5F). Based on the results described above, we determined that HF-MSCs inhibited pathological activation of JS1 cells and that activation of the TGF-β signaling pathway, as determined by Smad3 phosphorylation, was also suppressed in the HF-MSC group.

### HF-MSCs inhibited pathological HSC activation and Smad3 phosphorylation in the LC model

**Figure 6:**
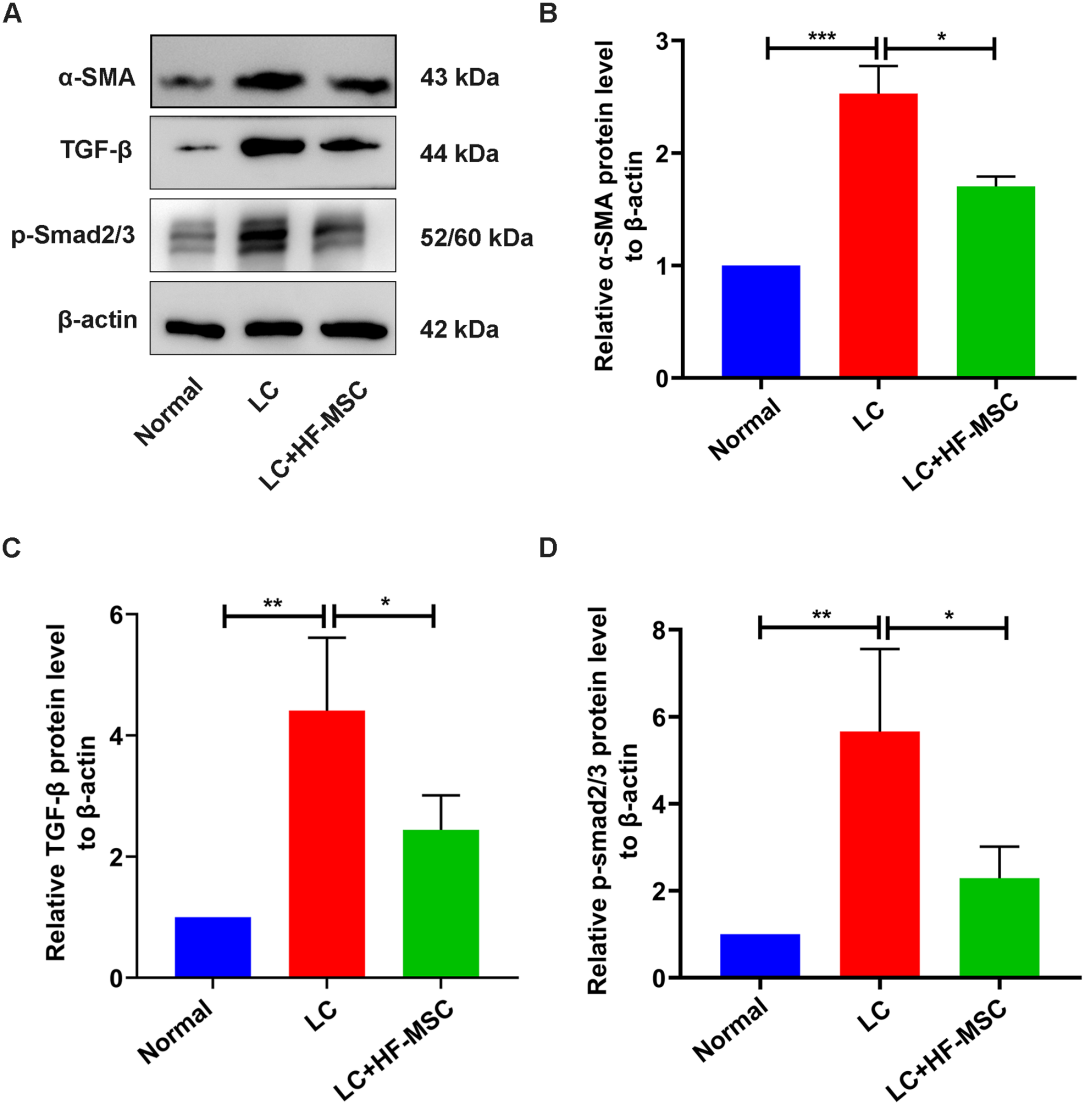
HF-MSCs inhibited HSC activation by inhibiting the TGF-β/Smad pathway *in vivo*. (A) The protein expression of α-SMA, TGF-β, and p-Smad2/3 in the three groups (*n* = 6). (B–D) Semiquantitative analysis of α-SMA, TGF-β, and p-Smad2/3. All data are shown as the means ± SD (One-way-ANOVA, *n* = 3). *P < 0.05, **P < 0.01, ***P < 0.001.

We next studied the role of HF-MSC treatment in regulating HSC activation. The expression of α-SMA, a marker of HSC activation, showed an increasing trend in the model group, which was reversed by HF-MSC treatment (*P* < 0.05) (Fig. 6). Furthermore, levels of the TGF-β and p-Smad3 proteins in the liver tissue were measured. Immunoblotting showed increased levels of TGF-β1 and p-Smad3 in the model group, but these changes were suppressed to a certain degree and approached the normal level in the HF-MSC treatment group (*P* < 0.05).

## Discussion

We proved that HF-MSCs have the ability to reverse LC, including ameliorating liver pathology and improving liver function. Additionally, transplanted HF-MSCs were recruited to inflamed liver tissues and differentiated into HLCs expressing hepatocyte-specific surface markers. In addition, HF-MSCs inhibited the pathological activation of HSCs by blocking the TGF-β/Smad pathway *in vitro* and *in vivo*.

CCl_4_ was used in our study to induce a mouse LC model because the liver injury caused by CCl_4_ is very similar to the pathological and functional changes observed in humans with cirrhosis (Marti-Rodrigo et al., 2020; Wu et al., 2020). CCl_4_ was administered at a low dose for a long period to ensure a low death rate in mice and the stability of the model. After 12 weeks of intraperitoneal injections of CCl_4_, the mice in the model group developed liver regeneration nodules and pseudolobules, and the serum levels of liver function markers indicating hepatocyte injury increased, indicating that we successfully established the LC model, as verified by the levels of liver serological indicators and liver pathology.

Decompensated cirrhosis has been reported to develop into hepatocyte carcinoma and hepatic failure with a poor prognosis. MSC therapy has been proven to have therapeutic potential in liver fibrosis/cirrhosis (Forbes & Newsome, 2016; Haldar et al., 2016; Zhang & Wang, 2013). The route of MSC infusion modulates the therapeutic effect of MSCs on LC. Three main methods have been developed to transplant MSCs, namely, intravenous injection, arterial injection, and local injection. Idriss et al. (2018) found that intravenous injection is a more appropriate and effective treatment for liver fibrosis than intrasplenic injection. Intravenous injection is commonly used in the clinic because of its convenience, and we also conducted cell therapy via tail vein injection in our research. After cell therapy for 3 weeks, liver function was restored to the greatest extent in the HF-MSC group, and we noticed that the deposition of collagen fibers assessed in liver pathological examinations was also reduced.

**Figure 7:**
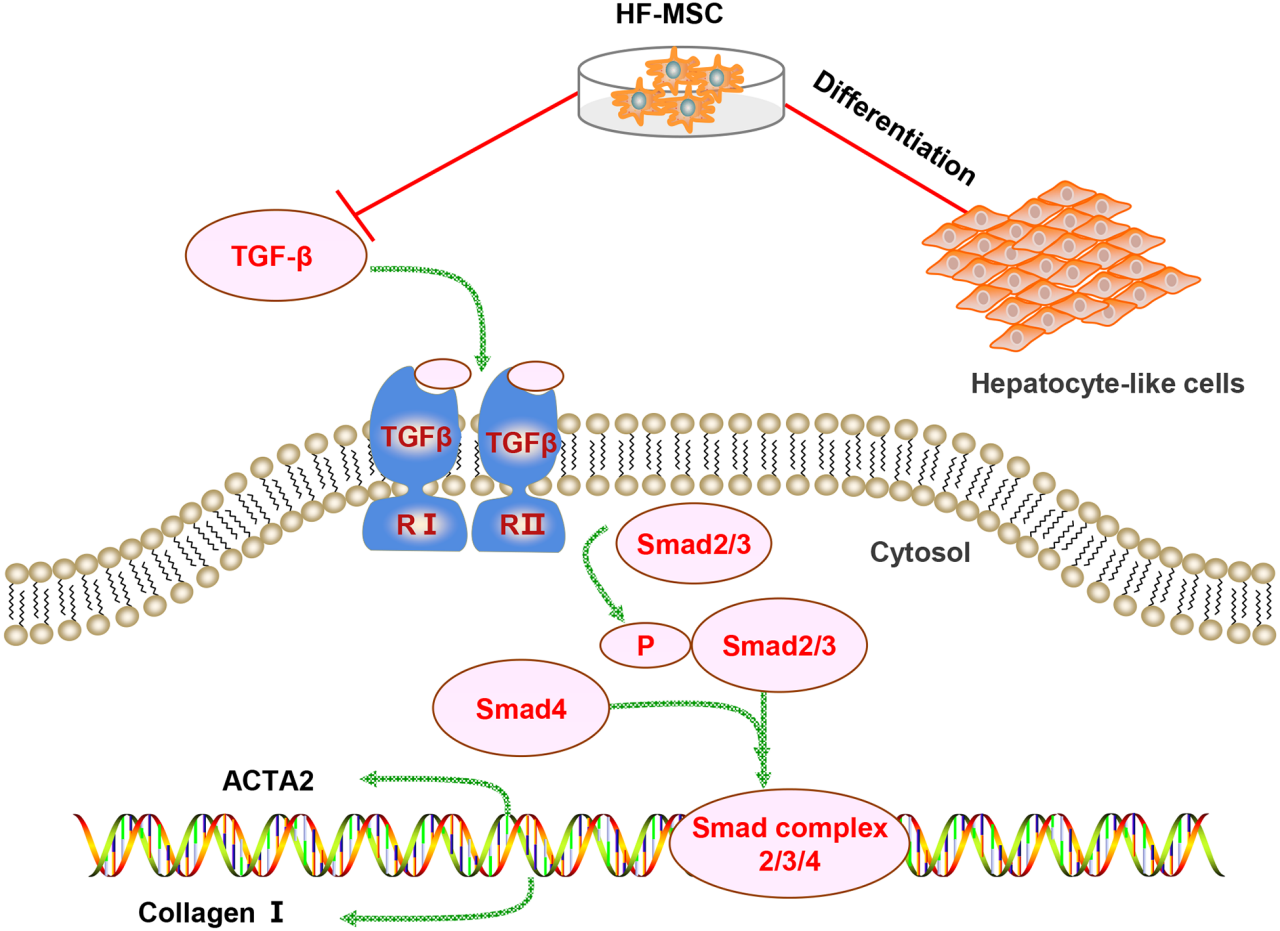
Diagram of the potential mechanism of the effects of HF-MSCs in the treatment of LC. The therapeutic effects of HF-MSCs on LC involves multiple mechanisms. On the one hand, they can migrate to the injured liver and differentiate into hepatocyte-like cells. On the other hand, HF-MSCs can inhibit the TGF-β/Smad pathway and thus inhibit the activation of HSCs, reducing collagen fibers and reversing cirrhosis.

Several mechanisms are involved in the therapeutic effect of HF-MSCs on LC. However, the differentiation of HF-MSCs and inhibition of pathological HSC activation may be involved in the therapeutic effect of HF-MSCs on liver injury (Fig. 7). First, the repair of liver injury requires a sufficient number of MSCs to home to the injured area. Infused MSCs have an increased implantation efficiency in damaged areas (Francois et al., 2006). Similarly, in our study, PKH67-labeled HF-MSCs homed to the injured liver and few HF-MSCs were observed in other organs. Although the mechanism of MSC homing has not been fully elucidated, some chemokines and adhesion factors are confirmed to be involved in MSC homing (Karp & Leng Teo, 2009). The mobilization and migration of MSCs are potentially mediated by the SDF-1/CXCR4 axis, which promote the migration and distribution of MSCs to the injured site (Yu et al., 2016). Second, the transplantation effect is associated with the number of homing MSCs (Karp & Leng Teo, 2009). Evidence has revealed that increasing the expression of E-selectin ligands on MSCs increases the homing of transplanted MSCs (Dykstra et al., 2016). Overall, an understanding of the homing mechanism and inducing more MSCs to home to the injured liver are worthy of further study.

MSCs can be induced into HLCs when they are exposed to the microenvironment of liver fibrosis (Alizadeh et al., 2016; Lin et al., 2010; Yang et al., 2021). As we expected, PKH67-stained HF-MSCs colocalized with the hepatocyte surface markers CK18 and ALB in our study, which confirms our hypothesis that HF-MSCs have the ability to differentiate into HLCs. The differentiation of MSCs is mediated by the local niche in the body. Therefore, the regulators that induce MSCs to transform into the liver lineage may be closely related to the liver environment. In addition, extensive cell–cell interactions are also involved in the process of inducing MSC differentiation into hepatocytes (Qihao et al., 2007). The mechanisms related to HF-MSC differentiation require further exploration to improve the efficiency of HF-MSC differentiation. Although the differentiation of MSCs into HLCs has been observed, the proportion of MSC-derived hepatocytes in the total liver mass is relatively low (Dai et al., 2009). Improving the ability of HF-MSCs to differentiate into HLCs contributes to the improvement of the effectiveness of HF-MSCs in treating LC. However, based on accumulating evidence, MSCs mainly exert antifibrotic effects through paracrine mechanisms (Berardis et al., 2015). Various types of factors are transported outwards in a divergent mode to complete the biological function of HF-MSCs (Bjerkvig et al., 2005; György et al., 2011). The role of MSC paracrine mechanism in LC needs to be further studied.

Four fates of pathologically activated HSCs have been identified, namely, the conversion of pathologically activated HSCs to resting HSCs, cell apoptosis, immune clearance, and cell senescence (Bataller & Brenner, 2005; Chu & Liaw, 2006). As the conversion of pathologically activated HSCs to myofibroblasts is the critical link in the development of LC (Kubo et al., 2015), inhibiting pathological HSC activation might represent an essential method to treat LC (Higashi, Friedman & Hoshida, 2017). In our research, after coculture with HF-MSCs *in vitro*, HF-MSCs inhibited the pathological activation of JS1 cells, as represented by α-SMA expression. A similar result was also obtained *in vivo*. Researchers have tried to determine how MSCs affect HSCs. The reported hypotheses include direct and indirect effects of MSCs on HSCs. Direct effects are mediated by the direct contact between the two cells or the cytokines secreted by MSCs on HSCs. MSCs can also indirectly affect the fate of HSCs by regulating hepatocytes and immune cells such as macrophages (Higashiyama et al., 2007; Maggini et al., 2010; Yu et al., 2015). Due to the excessive pathological activation of HSCs, phenotypic reversal is an effective method to inhibit the process of cirrhosis and improve the liver function of patients with cirrhosis. The regulatory effect of MSCs on HSCs provides strong support for the clinical application of MSCs. Clarifying the mechanism by which HF-MSCs inhibit HSC pathological activation would be helpful for identifying a more effective treatment for LC.

The pathological activation of HSCs is related to many factors, such as epithelial cell injury, changes in the extracellular matrix, TGF-β signal transduction, chronic infection with hepatitis virus, and intestinal dysfunction (Katsarou et al., 2020; Yavuz et al., 2020). Among these factors, TGF-β signal is a classical pathway leading to the occurrence of LC. TGF-β1 is protein that activates the TGF-β1/Smad pathway, which mediates pathological HSC activation and LC occurrence (Wu et al., 2017). The downstream signaling molecules of TGF-β1 are members the Smad protein family, which regulate collagen gene expression and target gene transcription. Blocking the TGF-β/Smad signaling pathway represents a powerful method to inhibit pathological HSC activation and alleviate LC. Xuan et al. (2017) proved that MSCs relieve hepatic injury by inhibiting the TGF-*β*1/Smad pathway. Consistent with their findings, the TGF-β signaling pathway was abnormally activated in mice with LC, while the levels of TGF-β and p-Smad2/3 were dramatically decreased in the HF-MSC group and tended to be normal in the present study. As shown in the present study, HF-MSCs inhibit pathological HSC activation and reverse LC by inhibiting the TGF-β1/Smad pathway.

## Conclusions

In summary, HF-MSCs migrate to the injured liver and differentiate into HLCs. HF-MSC therapy promotes the recovery of liver function and pathology in a model of cirrhosis by inhibiting pathological HSC activation through effects on the TGF-β1/Smad pathway. Our current study may provide a promising method to treat LC. Additional basic research is needed to clarify the underlying mechanism before this approach is translated the clinic.

## Supplemental Information

10.7717/peerj.12872/supp-1 Supplemental Information 1 Raw data for Figs. 1B–1D (B): Primary HF-MSCs migrated from the bulge area; (C): Second-generation HF-MSCs; (D): Adipogenic differentiation of HF-MSCs;

10.7717/peerj.12872/supp-2 Supplemental Information 2 Raw data for Figs. 1E–1G (E): Osteogenic differentiation of HF-MSCs; (F): HF-MSCs express the specific marker CK15 with immunofluorescence; (G): PKH67-labeled HF-MSCs show green light under a fluorescence microscope, and the nucleus is stained blue by DAPI. Scale bar (B–G): 50 µm

10.7717/peerj.12872/supp-3 Supplemental Information 3 Raw data for Figs. 2A–2B (A, B): HE and Masson staining between the normal group and the LC group. Scale bar (A, B): 50 µm

10.7717/peerj.12872/supp-4 Supplemental Information 4 Raw data for Figs. 2C–2E C–E: Changes in the levels of the serological indicators ALT, AST, and ALP in the normal and LC groups.

10.7717/peerj.12872/supp-5 Supplemental Information 5 Raw data for Figs. 3A–3B (A, B): HE staining and Masson staining in the three groups.

10.7717/peerj.12872/supp-6 Supplemental Information 6 Raw data for Figs. 3C–3E C–E: Comparison of liver serological indices ALT, AST, and ALB in the three groups.

10.7717/peerj.12872/supp-7 Supplemental Information 7 Raw data for Figs. 4A–4B Immunofluorescence staining-ALB

10.7717/peerj.12872/supp-8 Supplemental Information 8 Raw data for Figs. 4C–4D Immunofluorescence staining-ALB

10.7717/peerj.12872/supp-9 Supplemental Information 9 Raw data for Figs. 4E–4F Immunofluorescence staining-CK18

10.7717/peerj.12872/supp-10 Supplemental Information 10 Raw data for Figs. 4G–4H Immunofluorescence staining-CK18

10.7717/peerj.12872/supp-11 Supplemental Information 11 Raw data for Figs. 4I–4J Pearson’s correlation of liver sections costained with ALB and CK18.

10.7717/peerj.12872/supp-12 Supplemental Information 12 Raw data for Figs. 4K–4L PKH67-labeled HF-MSCs were rarely seen in the intestine, lung.

10.7717/peerj.12872/supp-13 Supplemental Information 13 Raw data for Figs. 4M–4N PKH67-labeled HF-MSCs were rarely seen in the spleen and kidney.

10.7717/peerj.12872/supp-14 Supplemental Information 14 Raw data for Figs. 5A–5B (A): Quiescent JS1 cells are in a static state, with irregular shapes, and the cell bodies are ovular or irregular; (B): The activated JS1 cells have enlarged cell bodies, protruding slender pseudopods, with a star-shaped appearance.

10.7717/peerj.12872/supp-15 Supplemental Information 15 Raw data for Fig5C-p-Smad2/3 The protein expression of p-smad23 and TGF-β

10.7717/peerj.12872/supp-16 Supplemental Information 16 Raw data for Fig. 5C-α-SMA-β-actin The protein expression of α-SMA and *β*-actin

10.7717/peerj.12872/supp-17 Supplemental Information 17 Raw data for Figs. 5D–5F Semiquantitative analysis of α-SMA, TGF-β, p-Smad2/3

10.7717/peerj.12872/supp-18 Supplemental Information 18 Raw data for Fig. 6A-p-smad23-TGF-β The protein expression of p-smad23 and TGF-β

10.7717/peerj.12872/supp-19 Supplemental Information 19 Raw data for Fig. 6A-α-SMA-β-actin The protein expression of α-SMA and *β*-actin

10.7717/peerj.12872/supp-20 Supplemental Information 20 Raw data for F Figs. 6B–6D Semiquantitative analysis of α-SMA, TGF-β, and p-Smad2/3

10.7717/peerj.12872/supp-21 Supplemental Information 21 ARRIVE 2.0 Checklist

## Additional Information and Declarations

### Data Availability

The following information was supplied regarding data availability: The raw measurements are available in the Supplementary Files.
