# Supplementary figures and images for: From hair to liver: emerging application of hair follicle mesenchymal stem cell transplantation reverses liver cirrhosis by blocking the TGF-β/Smad signaling pathway to inhibit pathological HSC activation
Source: PeerJ. 2022 Feb 15;10:e12872. doi: 10.7717/peerj.12872 (PMC8855721; doi:10.7717/peerj.12872)

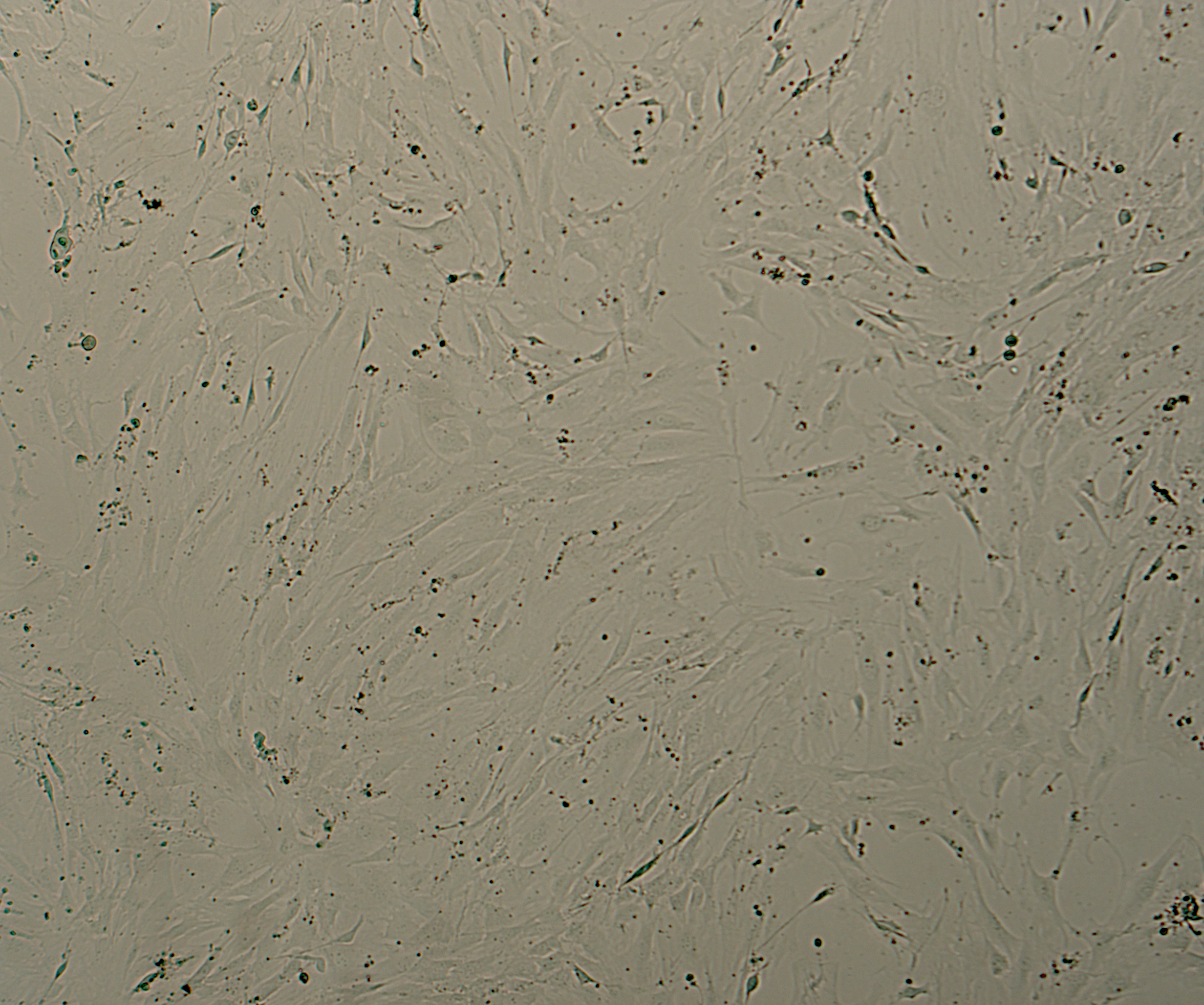

Supplement: Supplemental Information 1 — (B): Primary HF-MSCs migrated from the bulge area; (C): Second-generation HF-MSCs; (D): Adipogenic differentiation of HF-MSCs; [file peerj-10-12872-s001.zip › Fig. 1B-D/Fig. 1C.jpg]

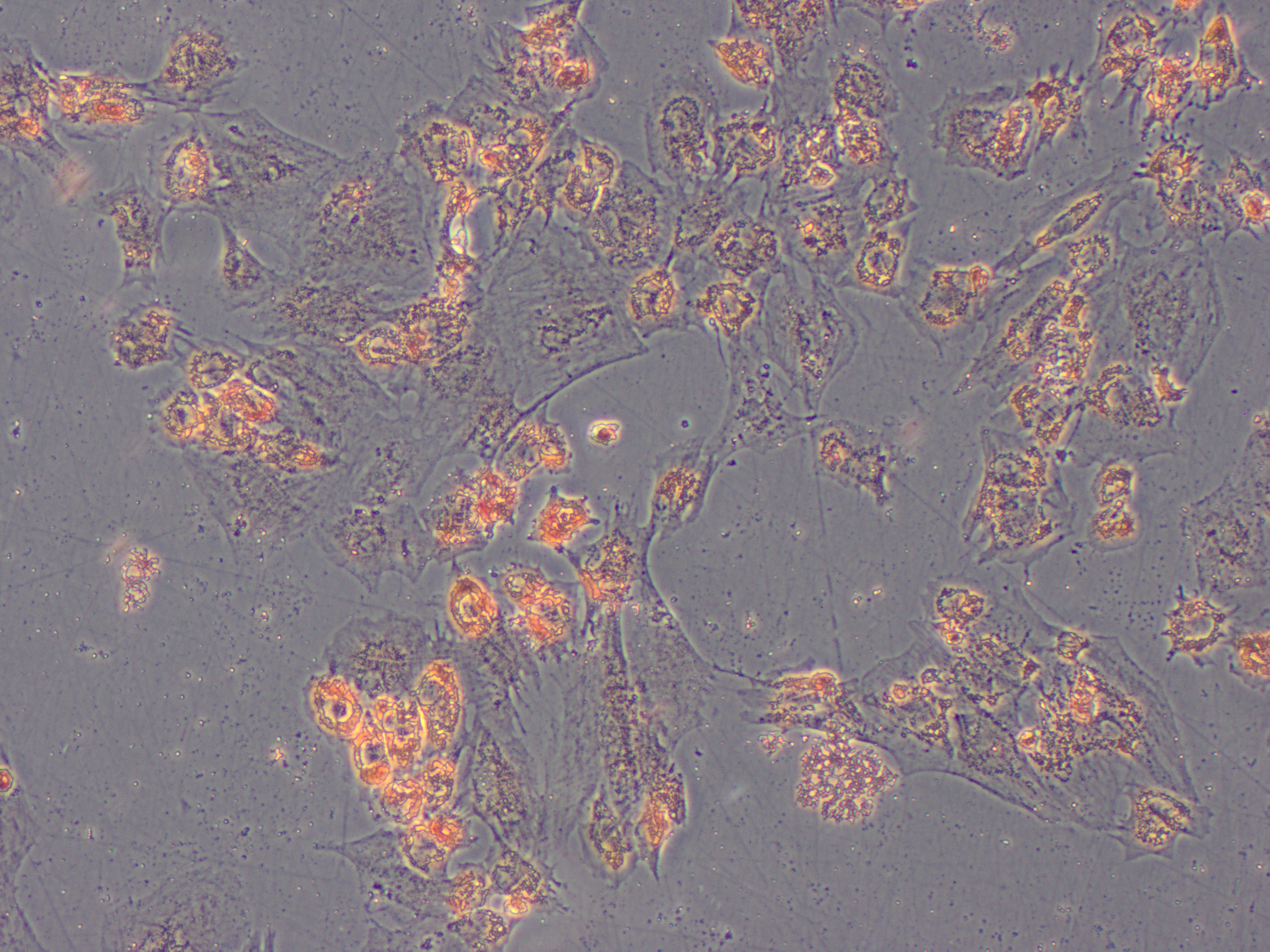

Supplement: Supplemental Information 1 — (B): Primary HF-MSCs migrated from the bulge area; (C): Second-generation HF-MSCs; (D): Adipogenic differentiation of HF-MSCs; [file peerj-10-12872-s001.zip › Fig. 1B-D/Fig. 1D.jpg]

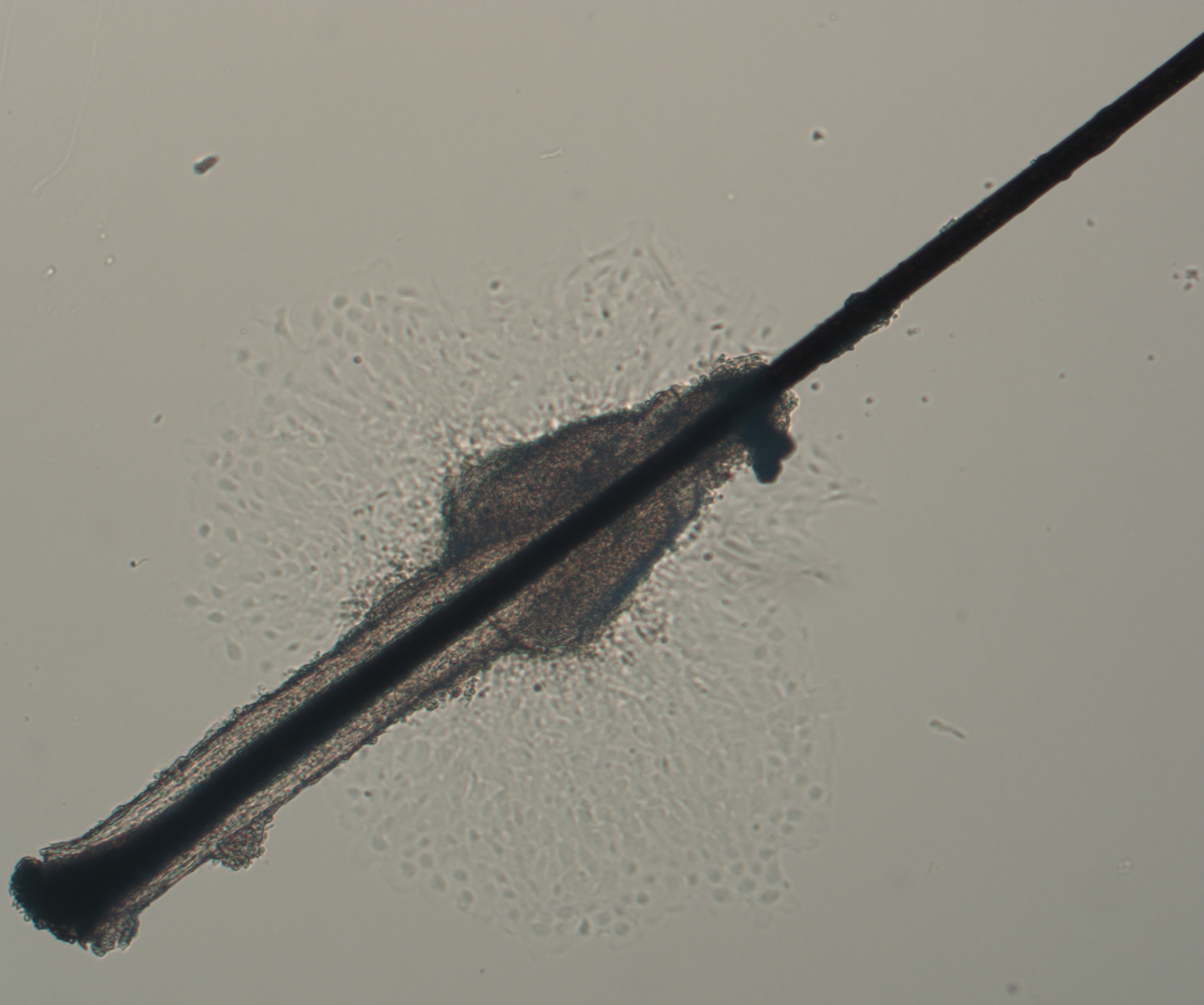

Supplement: Supplemental Information 1 — (B): Primary HF-MSCs migrated from the bulge area; (C): Second-generation HF-MSCs; (D): Adipogenic differentiation of HF-MSCs; [file peerj-10-12872-s001.zip › Fig. 1B-D/Fig.1B.jpg]

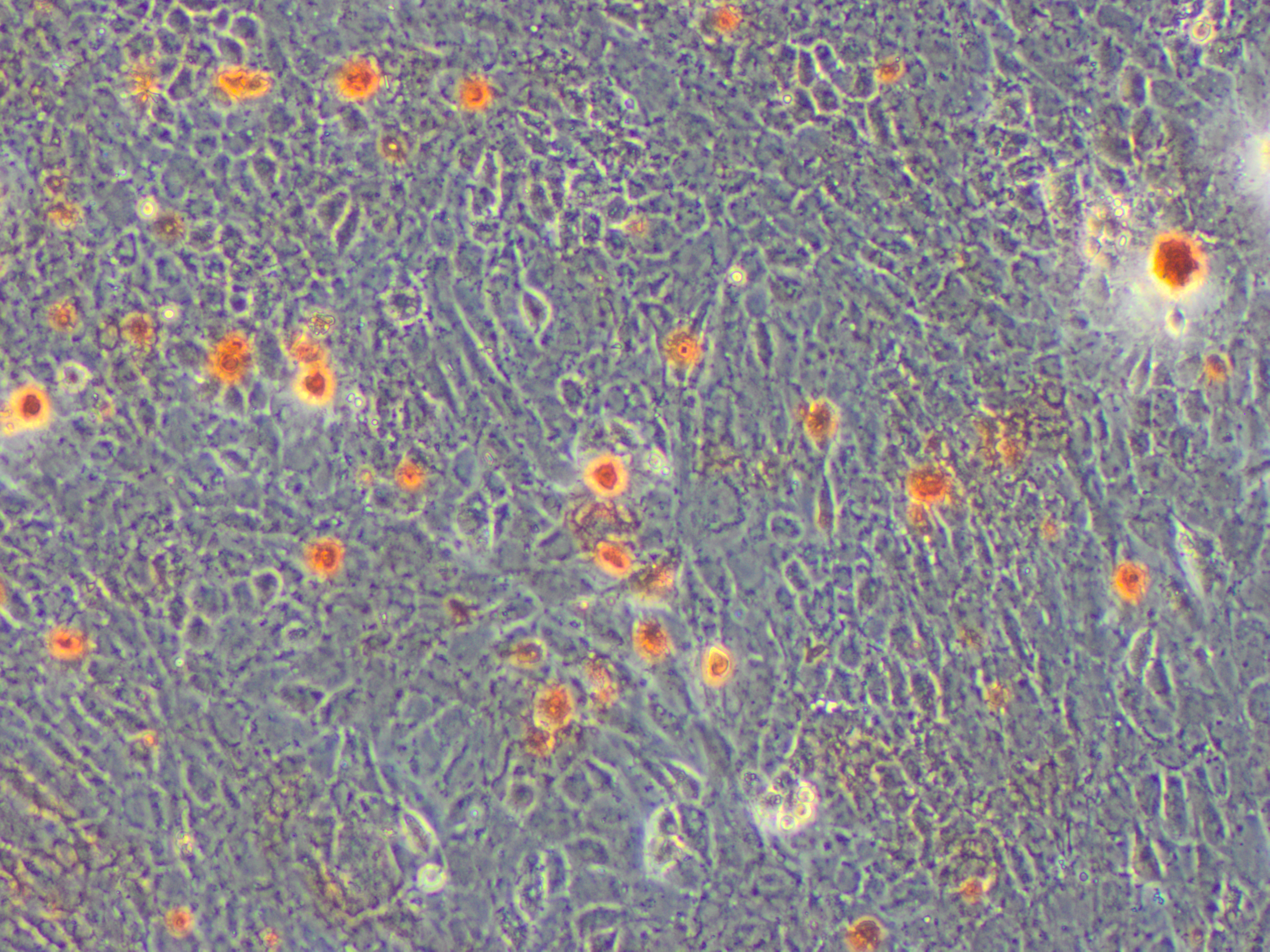

Supplement: Supplemental Information 2 — (E): Osteogenic differentiation of HF-MSCs; (F): HF-MSCs express the specific marker CK15 with immunofluorescence; (G): PKH67-labeled HF-MSCs show green light under a fluorescence microscope, and the nucleus is stained blue by DAPI. Scale bar (B–G): 50 µm [file peerj-10-12872-s002.zip › Fig. 1E-G/Fig. 1E.jpg]

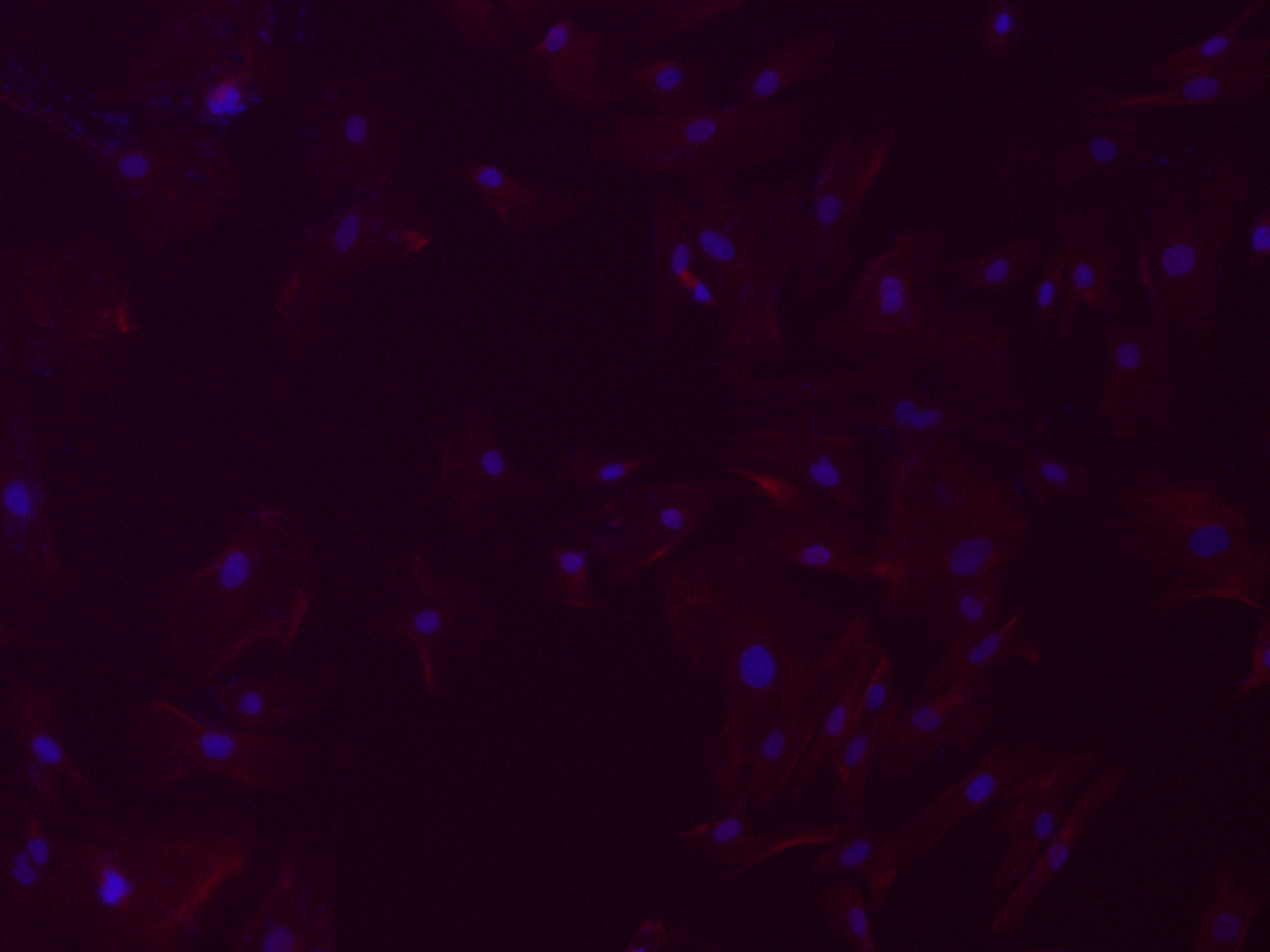

Supplement: Supplemental Information 2 — (E): Osteogenic differentiation of HF-MSCs; (F): HF-MSCs express the specific marker CK15 with immunofluorescence; (G): PKH67-labeled HF-MSCs show green light under a fluorescence microscope, and the nucleus is stained blue by DAPI. Scale bar (B–G): 50 µm [file peerj-10-12872-s002.zip › Fig. 1E-G/Fig. 1F.jpg]

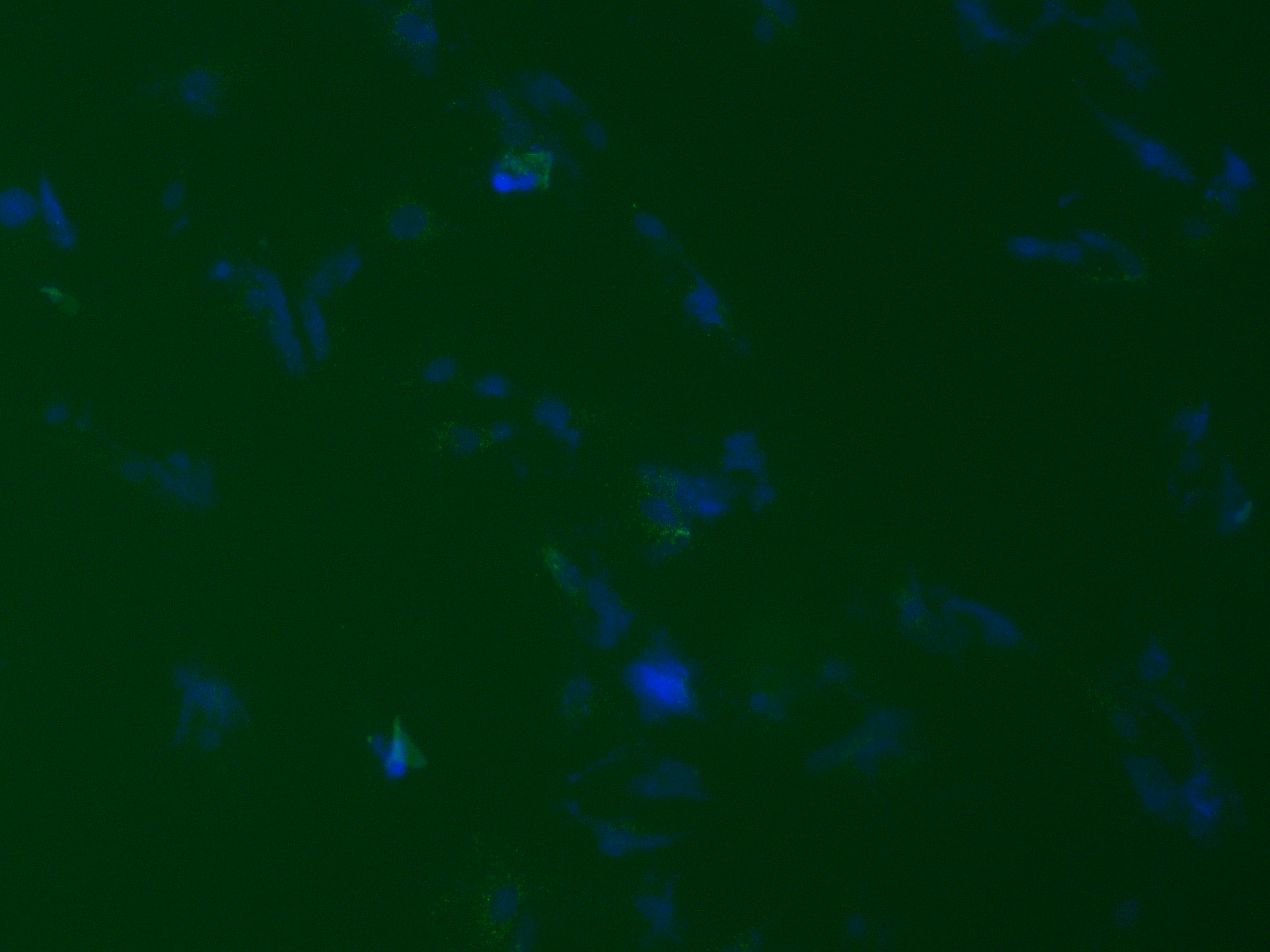

Supplement: Supplemental Information 2 — (E): Osteogenic differentiation of HF-MSCs; (F): HF-MSCs express the specific marker CK15 with immunofluorescence; (G): PKH67-labeled HF-MSCs show green light under a fluorescence microscope, and the nucleus is stained blue by DAPI. Scale bar (B–G): 50 µm [file peerj-10-12872-s002.zip › Fig. 1E-G/Fig. 1G.jpg]

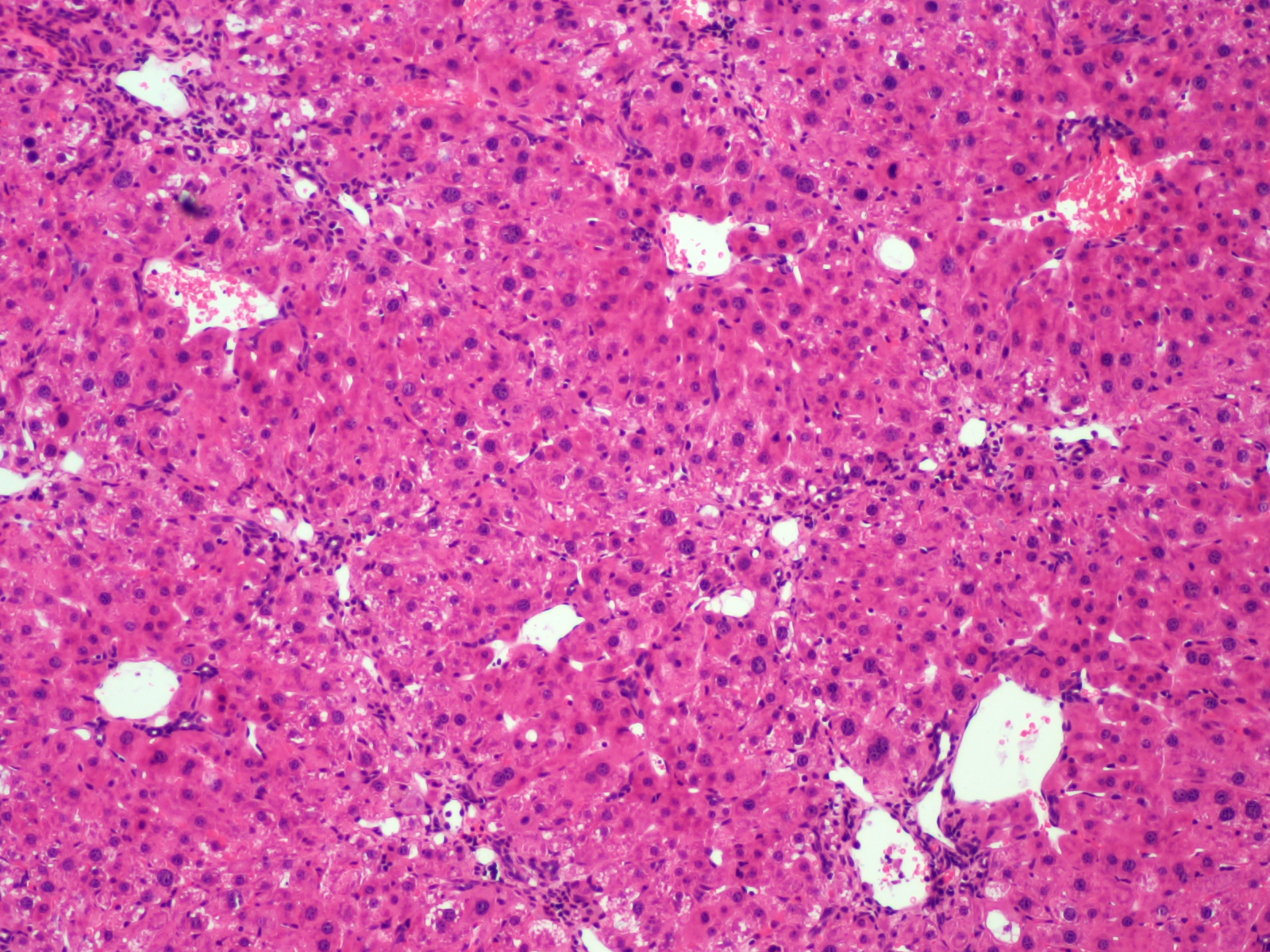

Supplement: Supplemental Information 3 — (A, B): HE and Masson staining between the normal group and the LC group. Scale bar (A, B): 50 µm [file peerj-10-12872-s003.zip › Fig. 2A-B/Fig. 2A-LC.jpg]

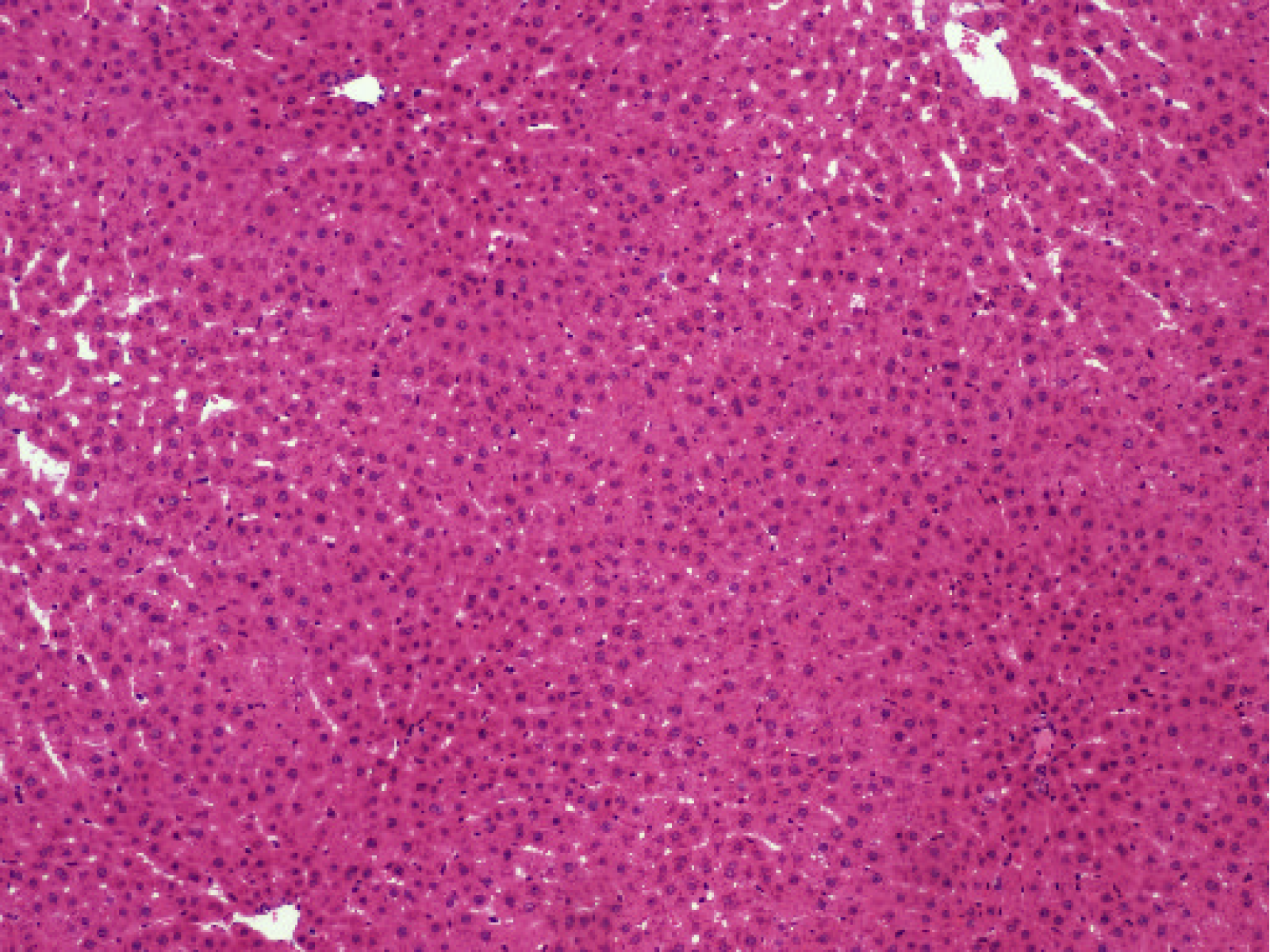

Supplement: Supplemental Information 3 — (A, B): HE and Masson staining between the normal group and the LC group. Scale bar (A, B): 50 µm [file peerj-10-12872-s003.zip › Fig. 2A-B/Fig. 2A-Normal.jpg]

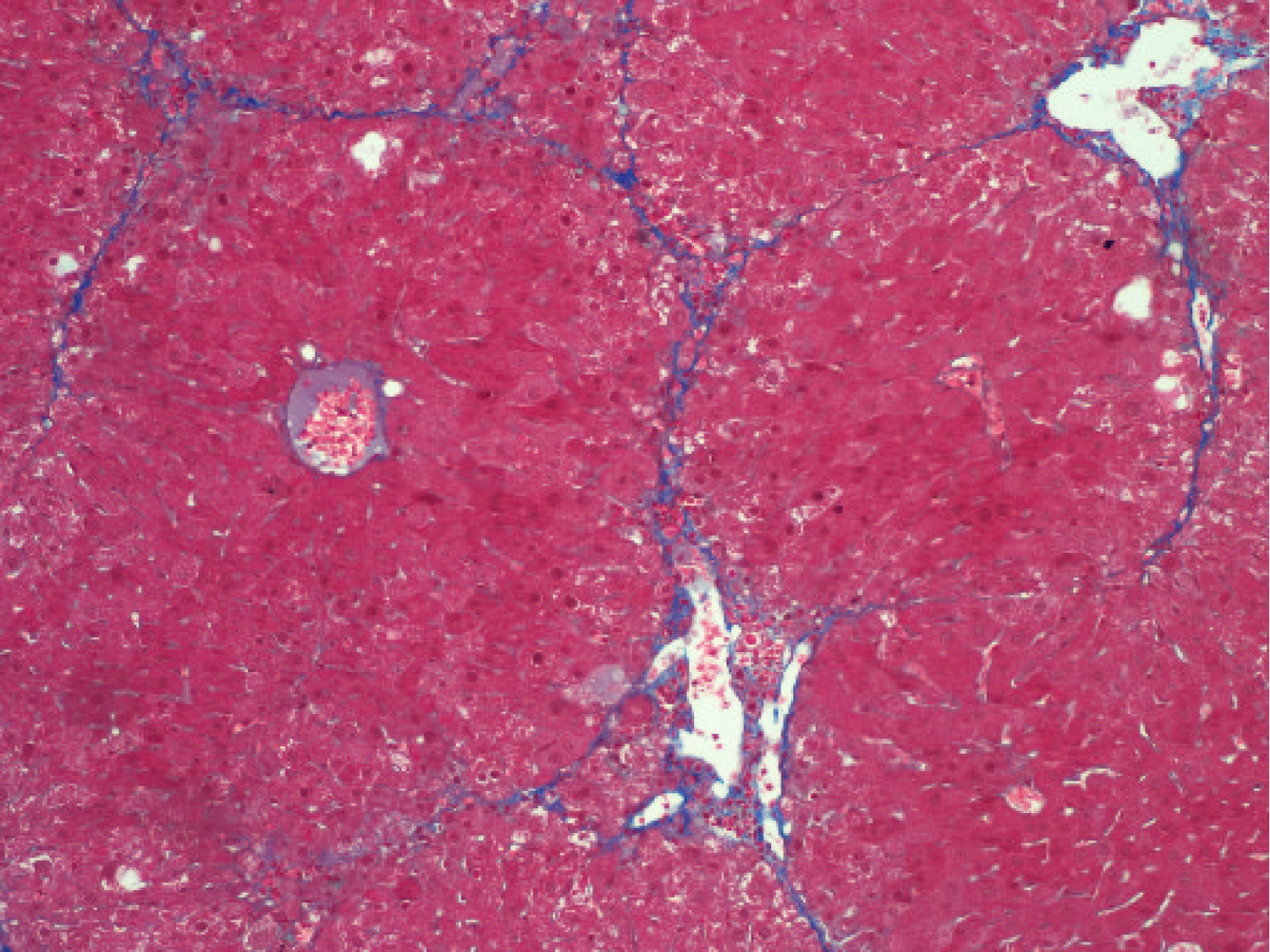

Supplement: Supplemental Information 3 — (A, B): HE and Masson staining between the normal group and the LC group. Scale bar (A, B): 50 µm [file peerj-10-12872-s003.zip › Fig. 2A-B/Fig. 2B-LC.jpg]

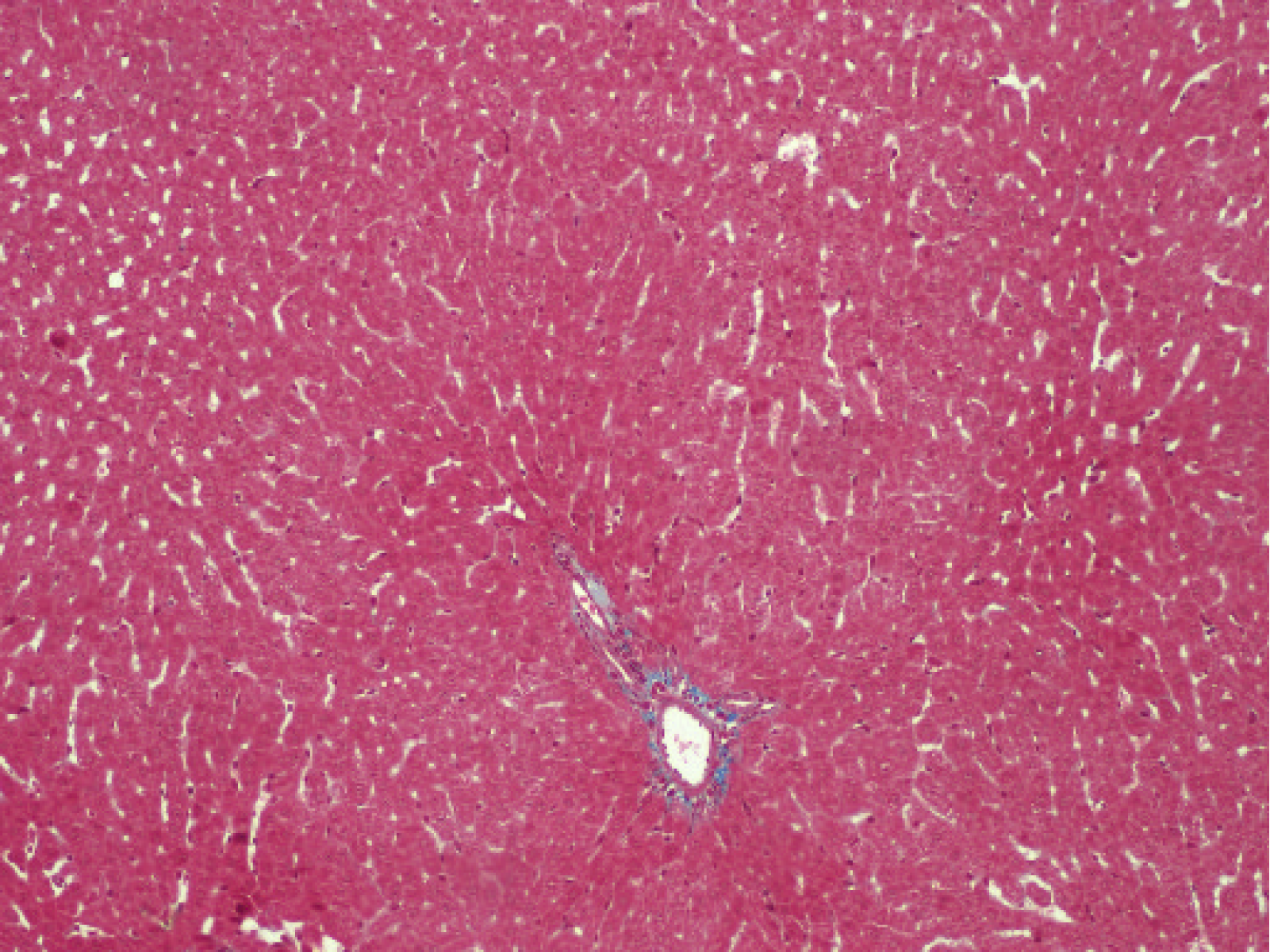

Supplement: Supplemental Information 3 — (A, B): HE and Masson staining between the normal group and the LC group. Scale bar (A, B): 50 µm [file peerj-10-12872-s003.zip › Fig. 2A-B/Fig. 2B-Noraml.jpg]

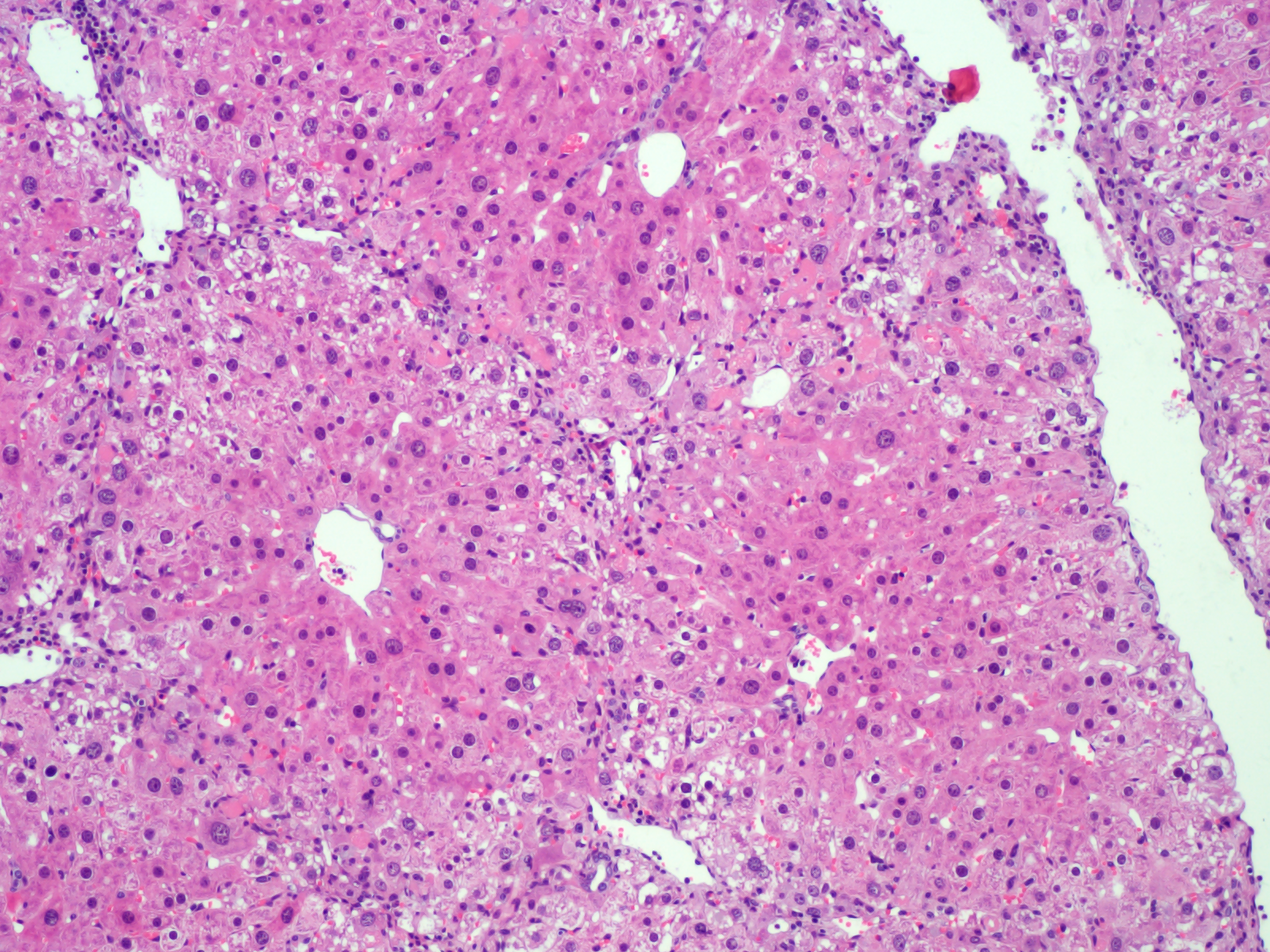

Supplement: Supplemental Information 5 — (A, B): HE staining and Masson staining in the three groups. [file peerj-10-12872-s005.zip › Fig. 3A-B/Fig. 3A-LC.jpg]

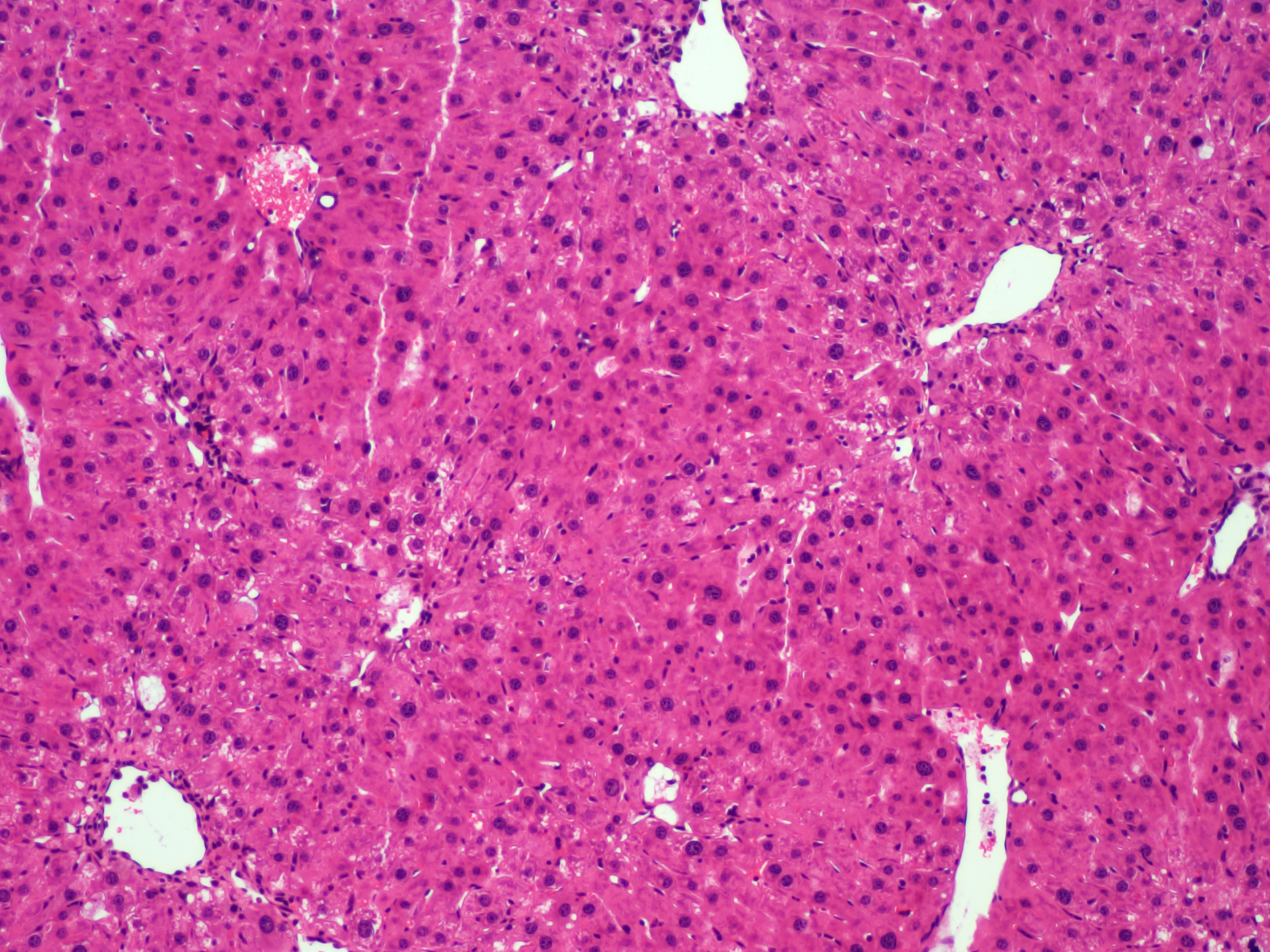

Supplement: Supplemental Information 5 — (A, B): HE staining and Masson staining in the three groups. [file peerj-10-12872-s005.zip › Fig. 3A-B/Fig. 3A-LC+HF-MSCs.jpg]

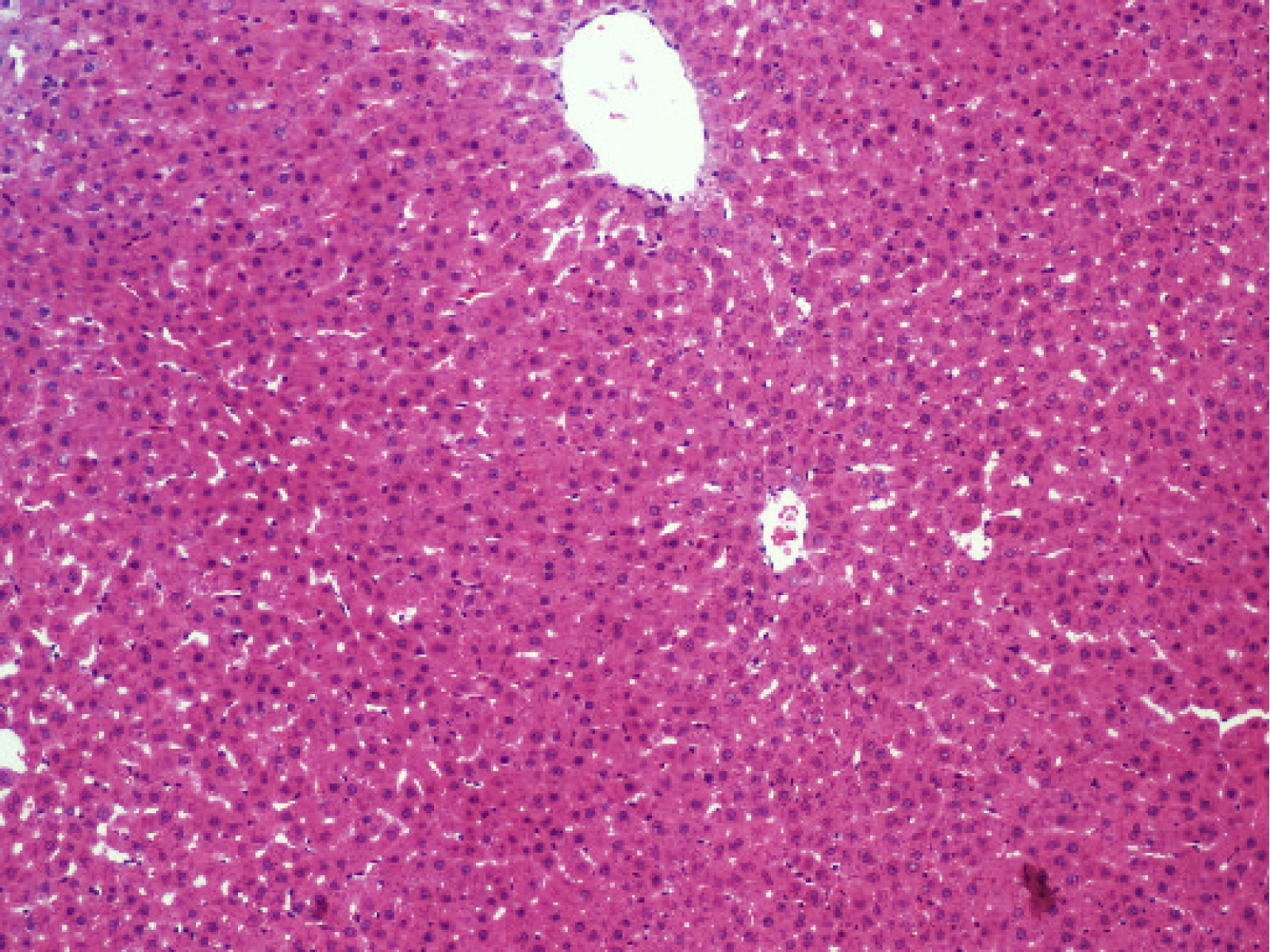

Supplement: Supplemental Information 5 — (A, B): HE staining and Masson staining in the three groups. [file peerj-10-12872-s005.zip › Fig. 3A-B/Fig. 3A-Normal.jpg]

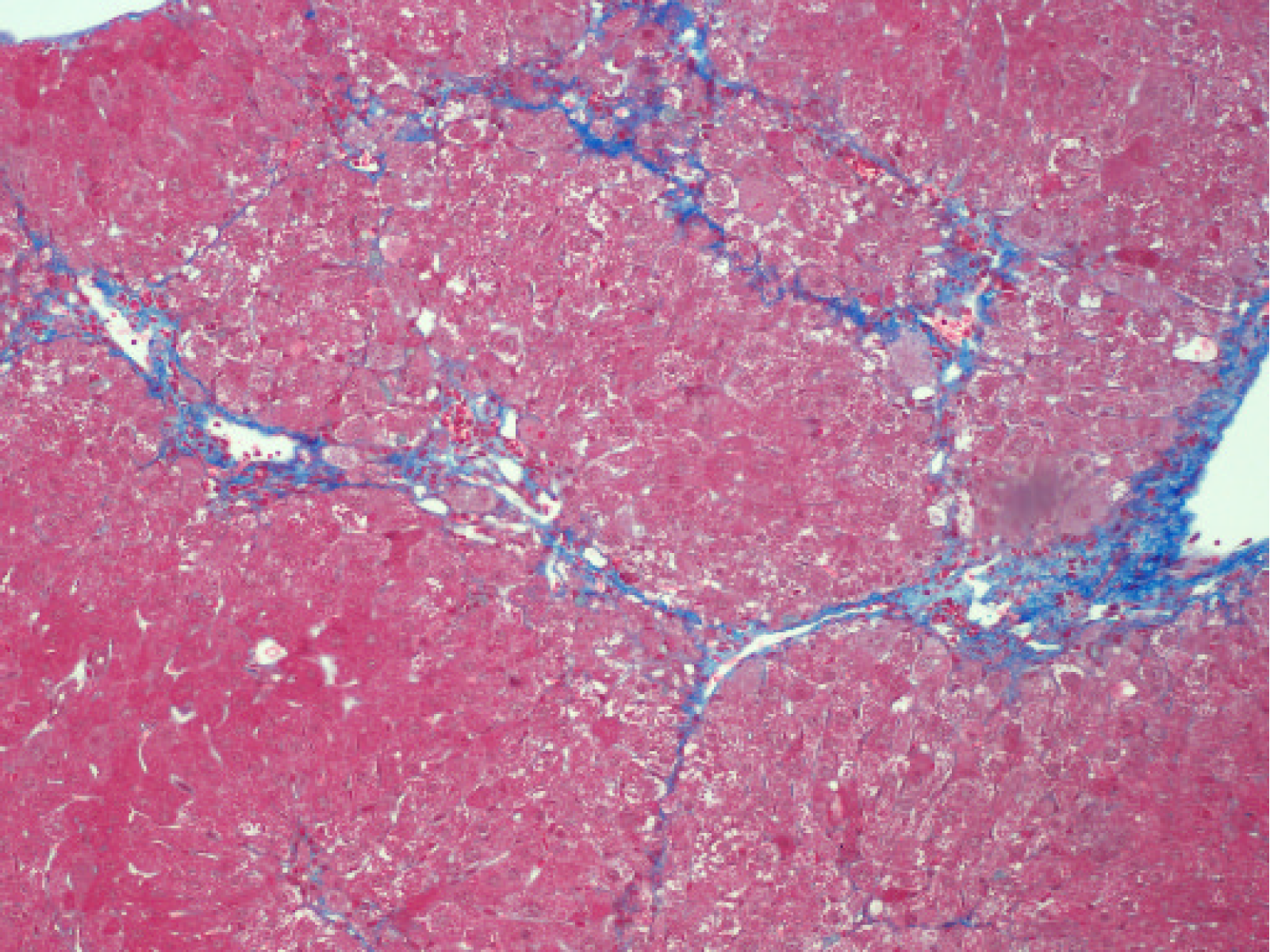

Supplement: Supplemental Information 5 — (A, B): HE staining and Masson staining in the three groups. [file peerj-10-12872-s005.zip › Fig. 3A-B/Fig. 3B-LC.jpg]

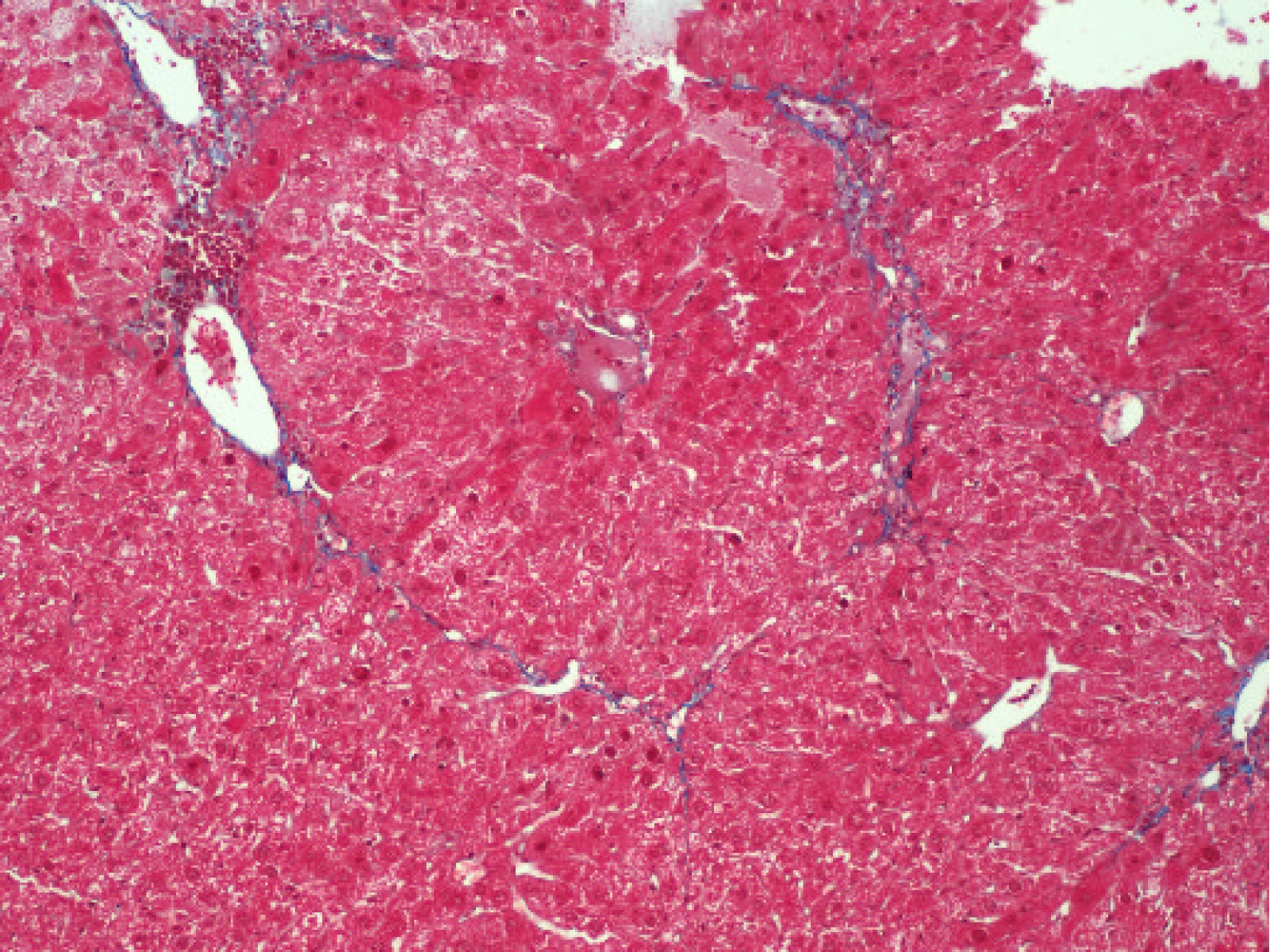

Supplement: Supplemental Information 5 — (A, B): HE staining and Masson staining in the three groups. [file peerj-10-12872-s005.zip › Fig. 3A-B/Fig. 3B-LC+HF-MSCs.jpg]

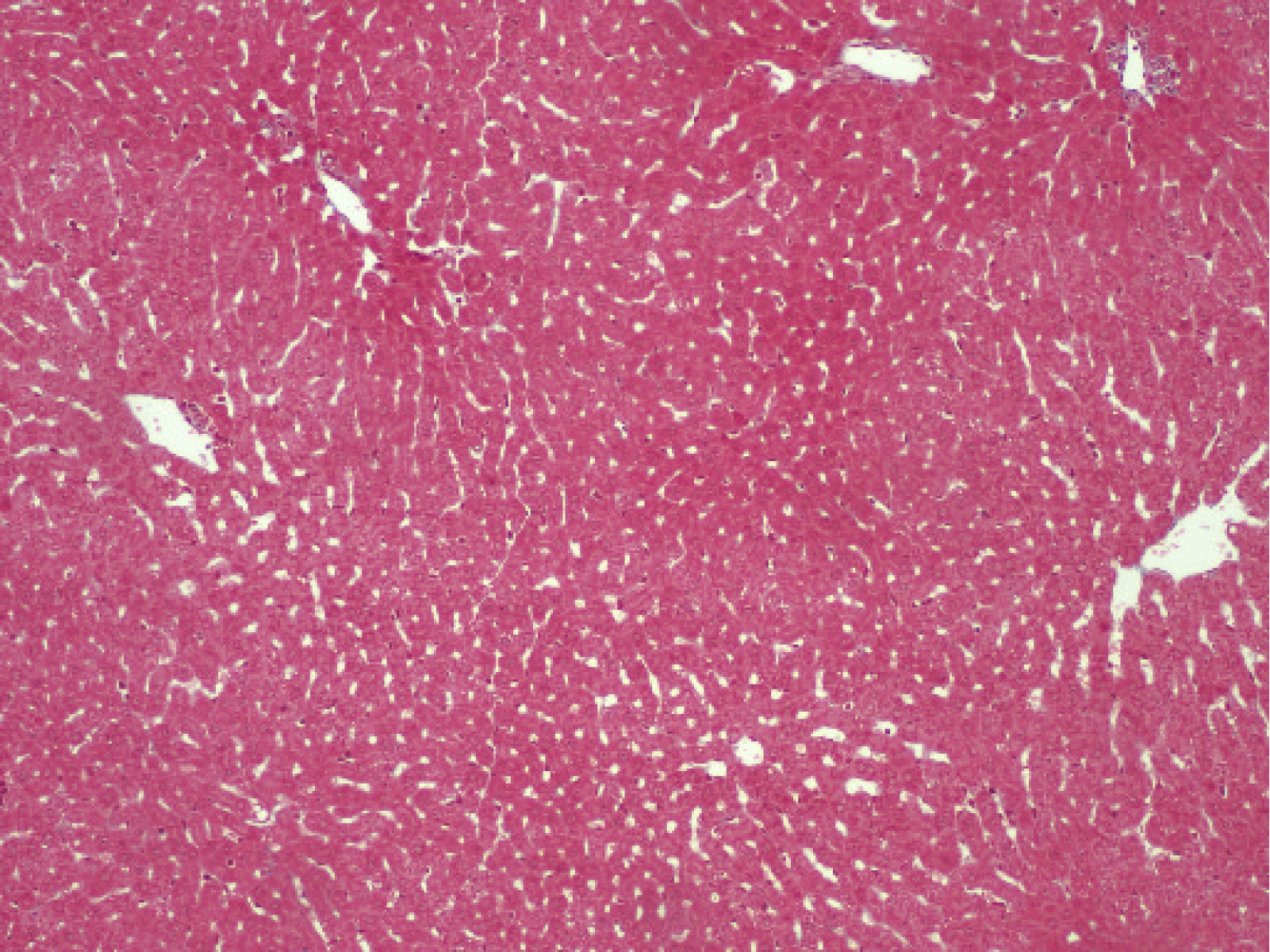

Supplement: Supplemental Information 5 — (A, B): HE staining and Masson staining in the three groups. [file peerj-10-12872-s005.zip › Fig. 3A-B/Fig. 3B-Normal.jpg]

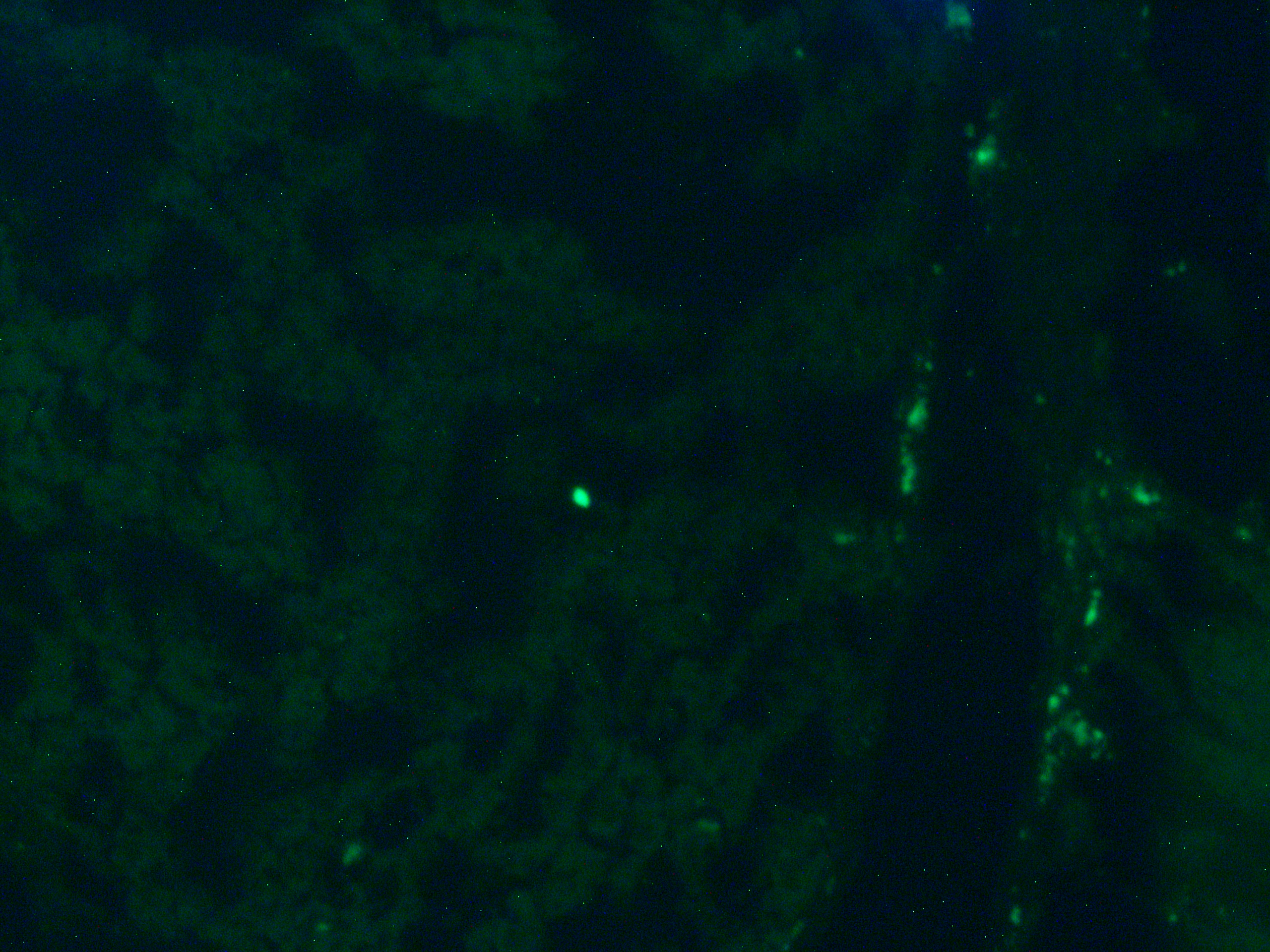

Supplement: Supplemental Information 7 — Immunofluorescence staining-ALB [file peerj-10-12872-s007.zip › Fig. 4A-B/Fig. 4A.jpg]

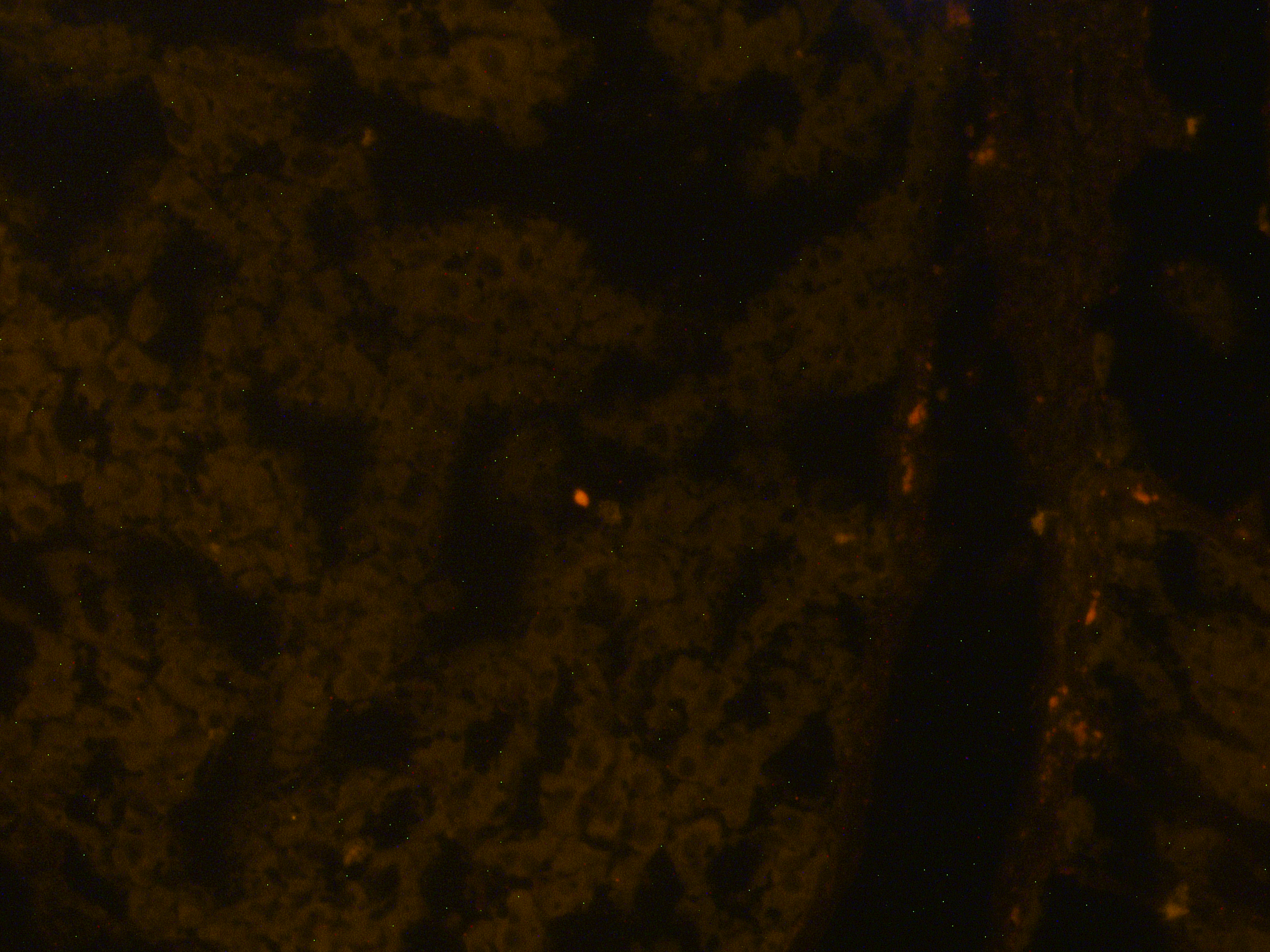

Supplement: Supplemental Information 7 — Immunofluorescence staining-ALB [file peerj-10-12872-s007.zip › Fig. 4A-B/Fig. 4B.jpg]

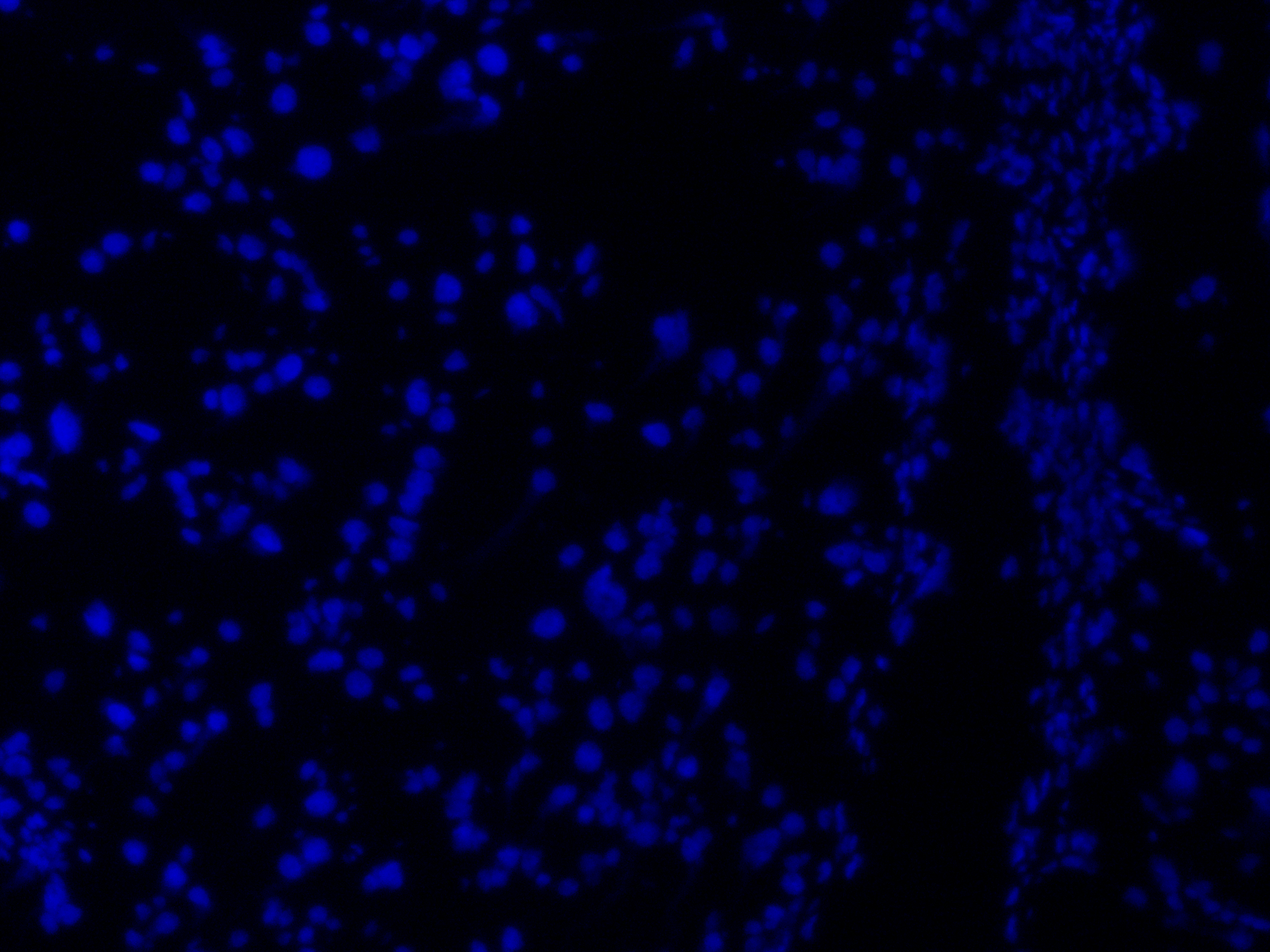

Supplement: Supplemental Information 8 — Immunofluorescence staining-ALB [file peerj-10-12872-s008.zip › Fig. 4C-D/Fig. 4C.jpg]

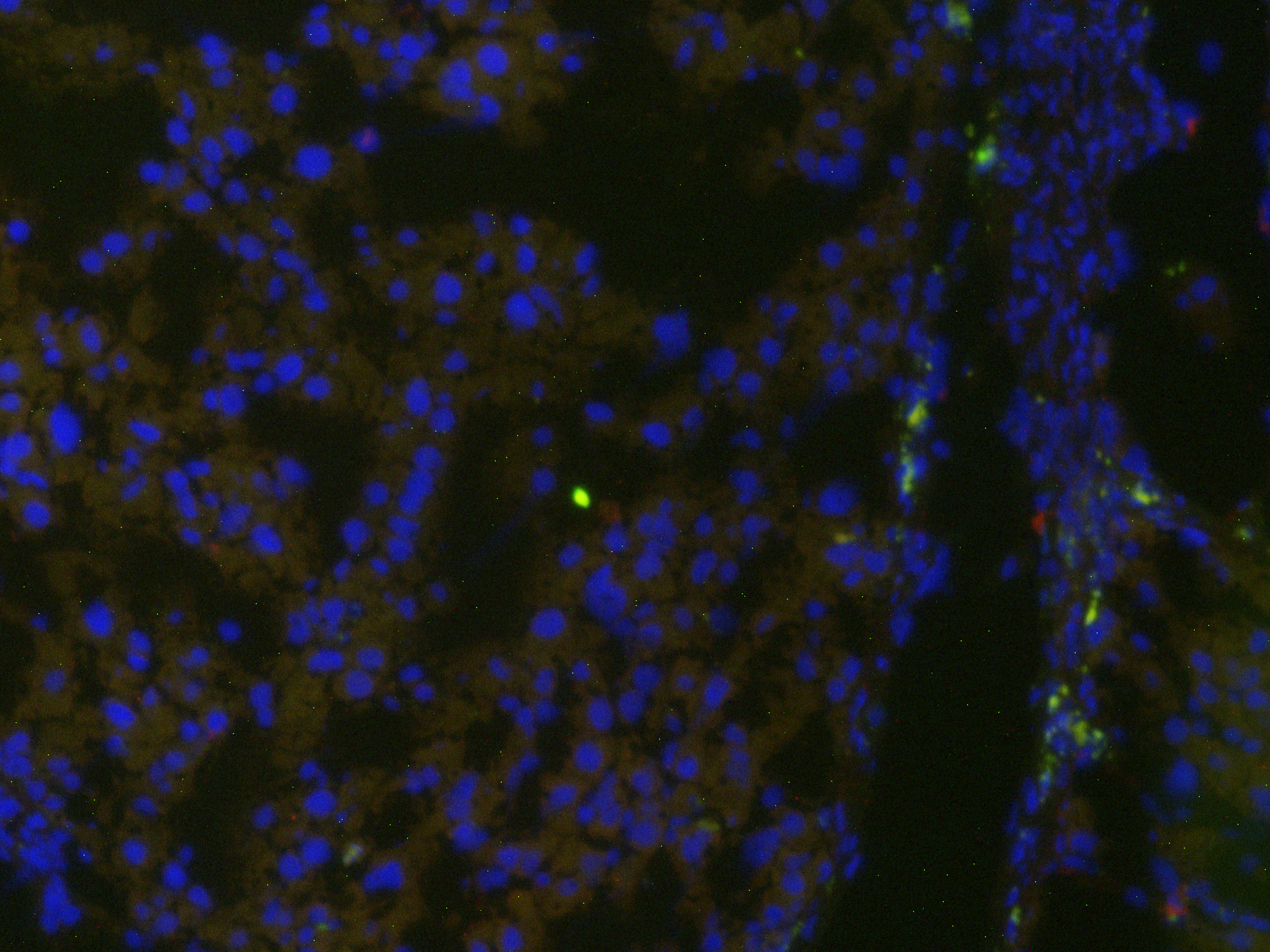

Supplement: Supplemental Information 8 — Immunofluorescence staining-ALB [file peerj-10-12872-s008.zip › Fig. 4C-D/Fig. 4D.jpg]

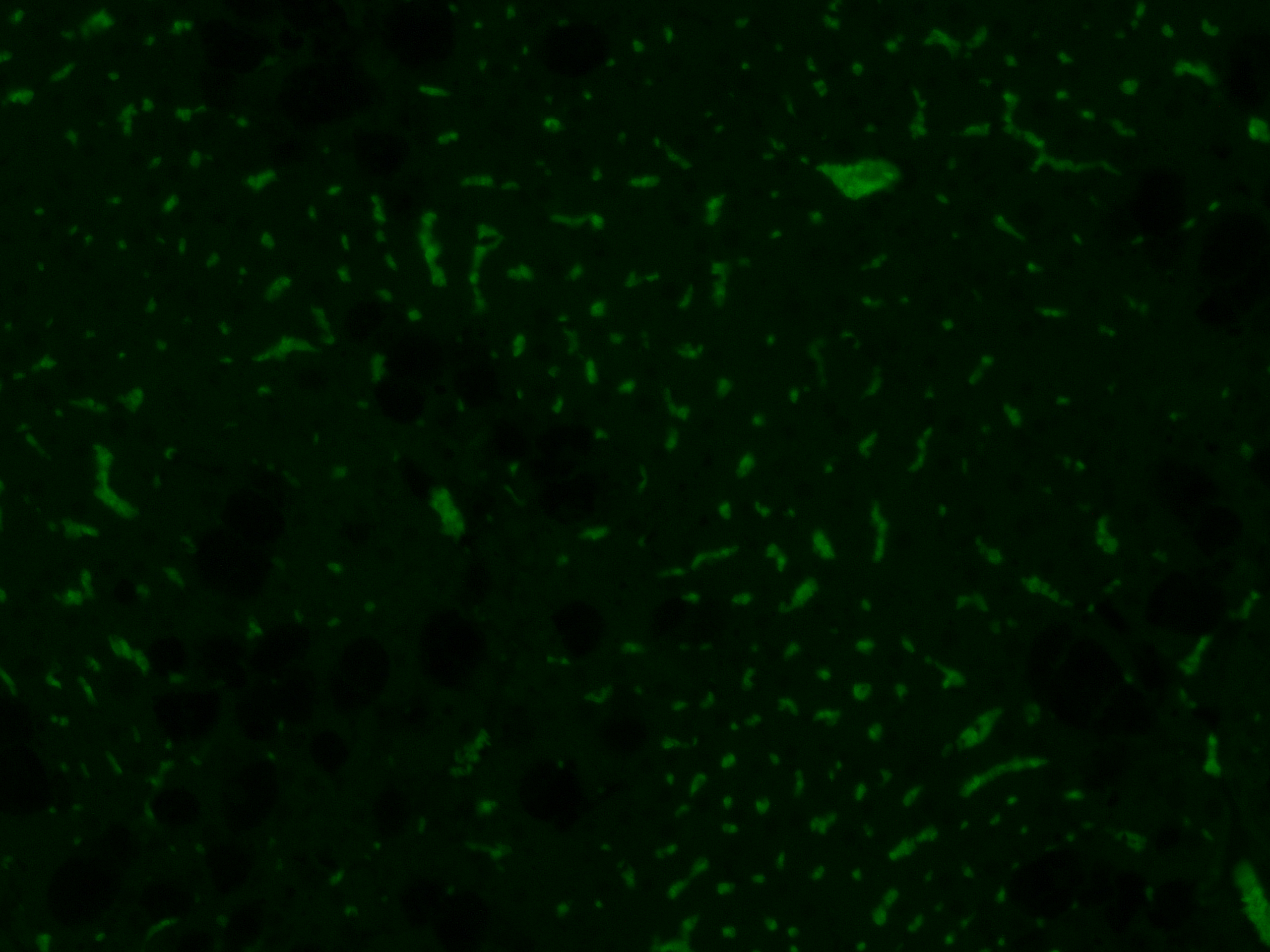

Supplement: Supplemental Information 9 — Immunofluorescence staining-CK18 [file peerj-10-12872-s009.zip › Fig. 4E-F/Fig. 4E.jpg]

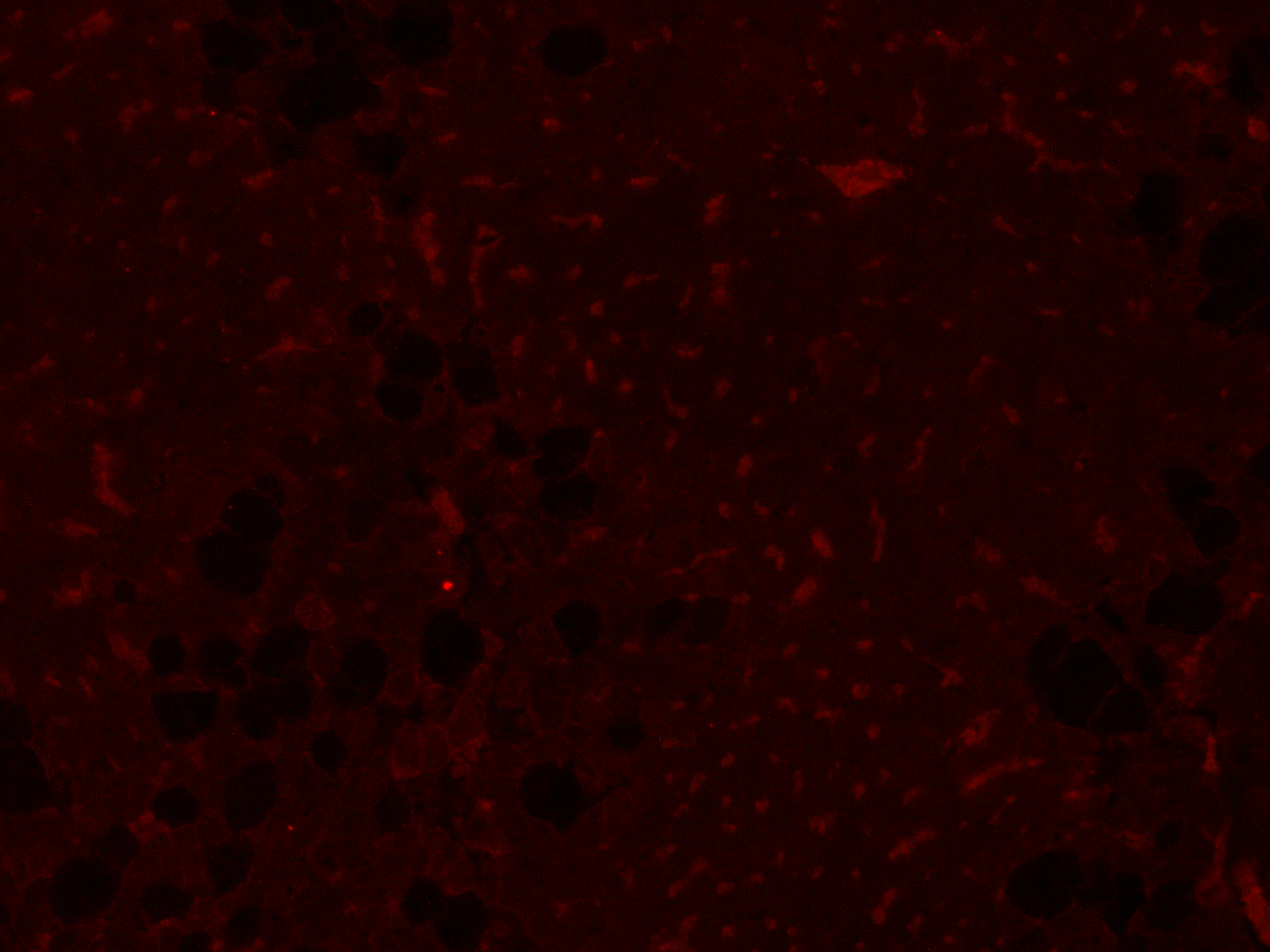

Supplement: Supplemental Information 9 — Immunofluorescence staining-CK18 [file peerj-10-12872-s009.zip › Fig. 4E-F/Fig. 4F.jpg]

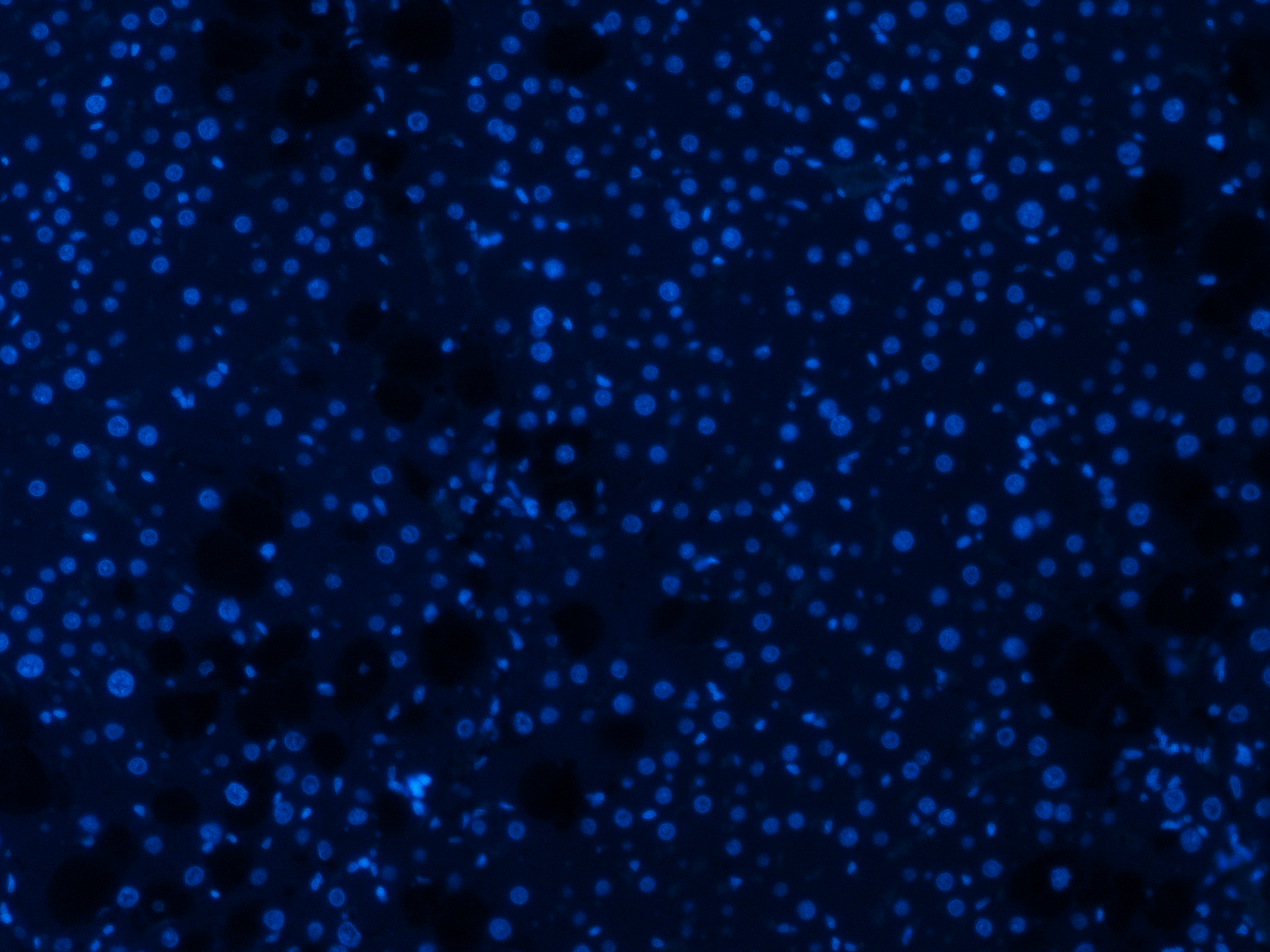

Supplement: Supplemental Information 10 — Immunofluorescence staining-CK18 [file peerj-10-12872-s010.zip › Fig. 4G-H/Fig. 4G.jpg]

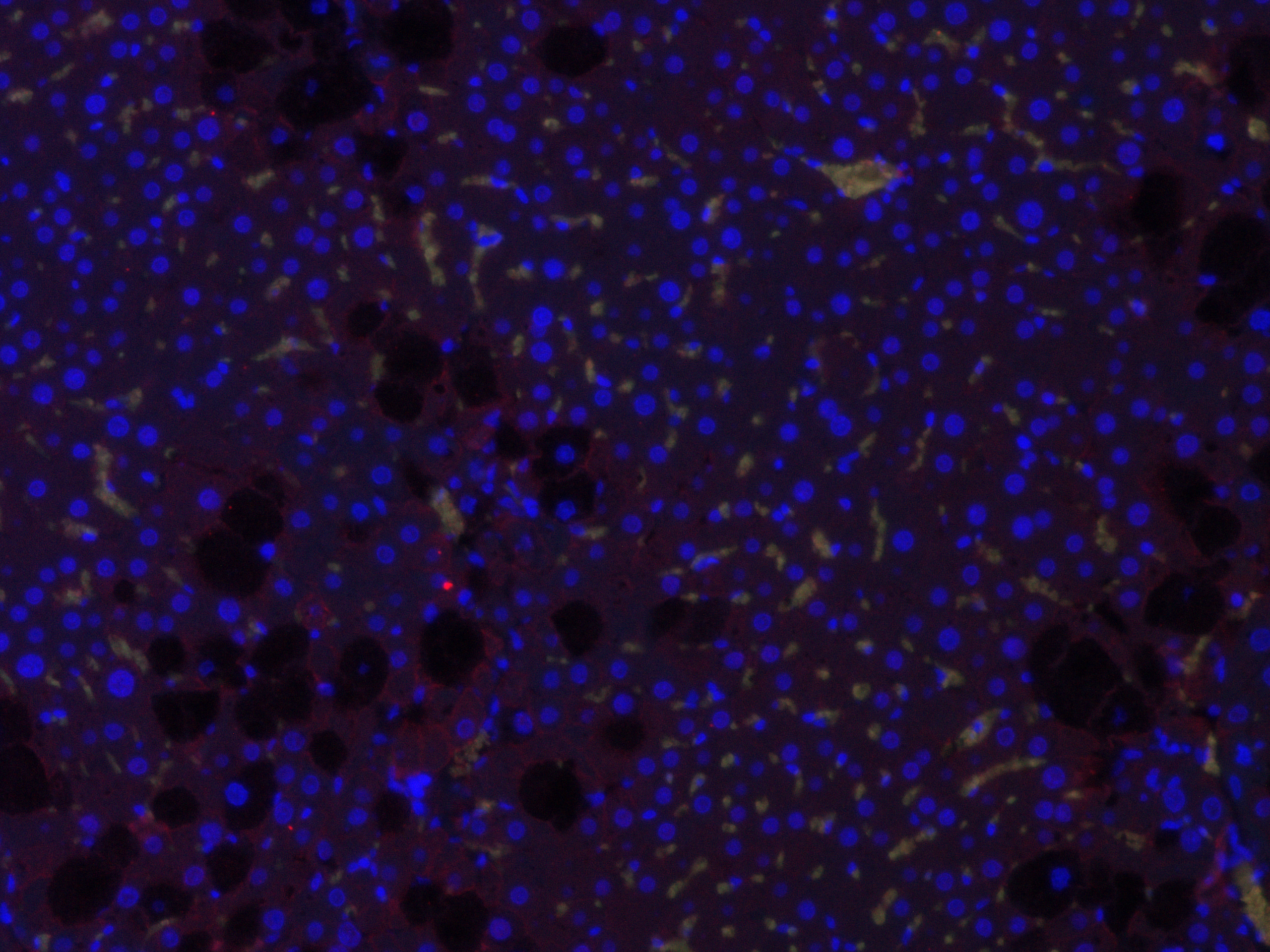

Supplement: Supplemental Information 10 — Immunofluorescence staining-CK18 [file peerj-10-12872-s010.zip › Fig. 4G-H/Fig. 4H.jpg]

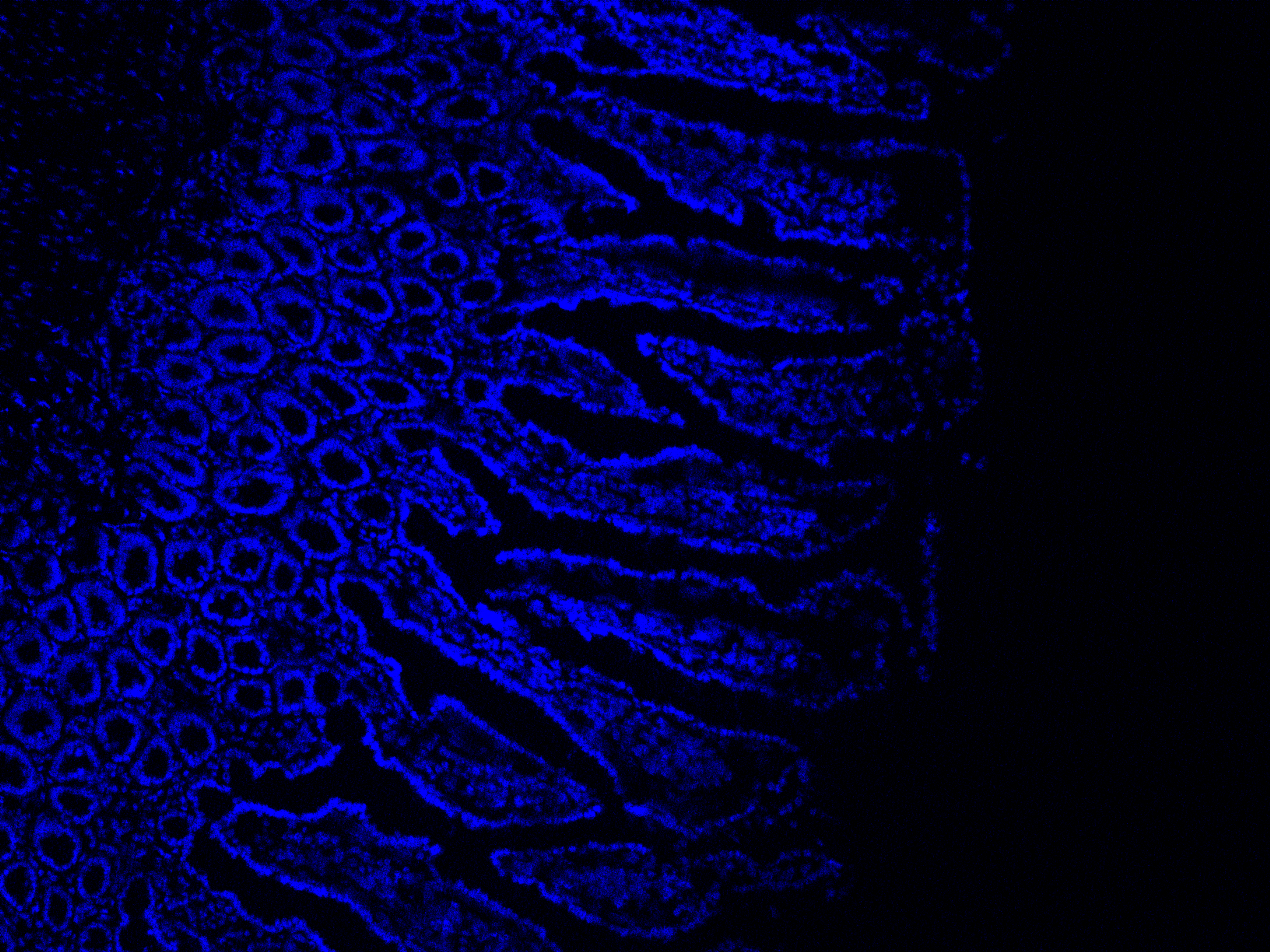

Supplement: Supplemental Information 12 — PKH67-labeled HF-MSCs were rarely seen in the intestine, lung. [file peerj-10-12872-s012.zip › Fig. 4K-L/Fig. 4K.jpg]

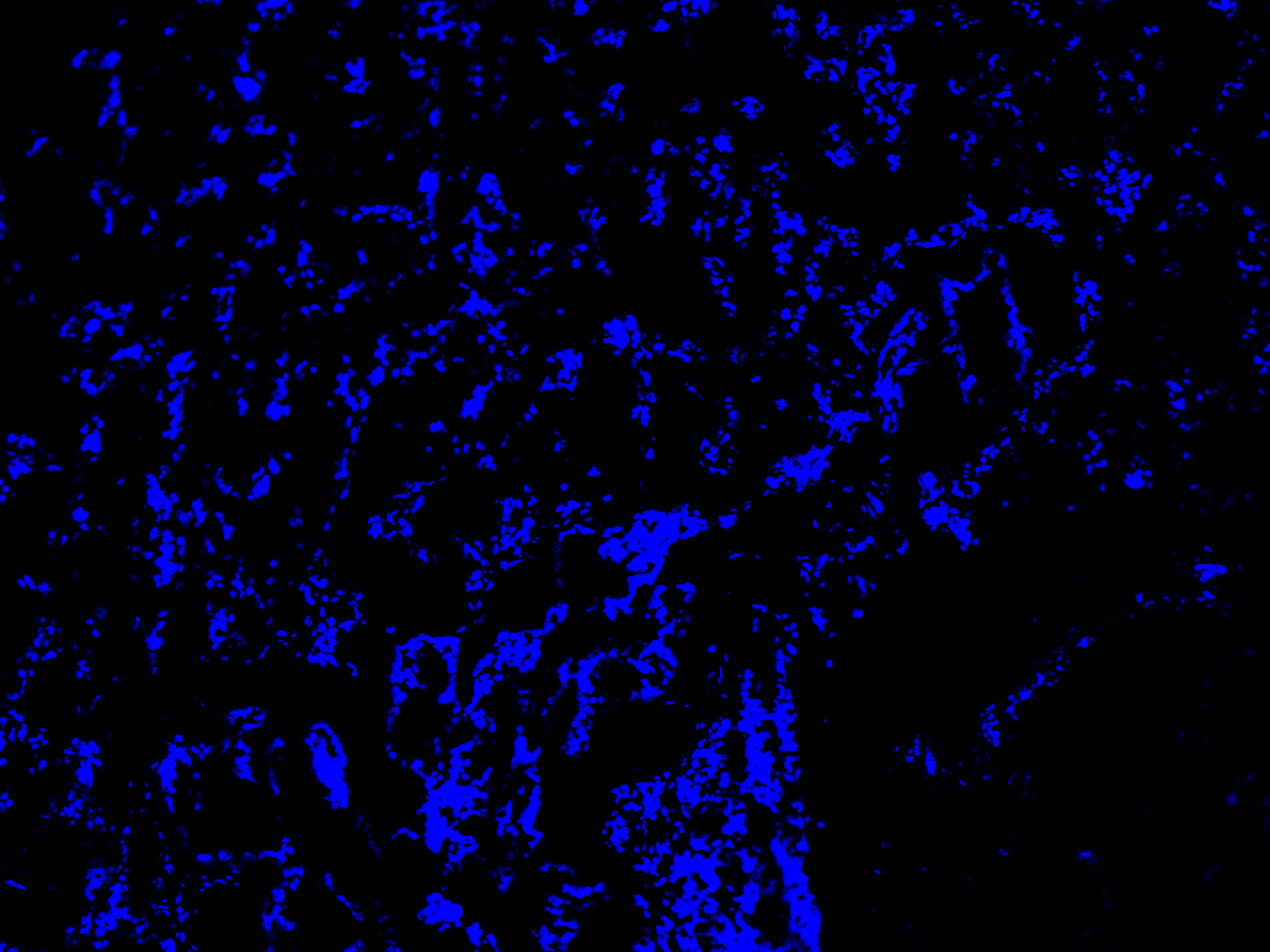

Supplement: Supplemental Information 12 — PKH67-labeled HF-MSCs were rarely seen in the intestine, lung. [file peerj-10-12872-s012.zip › Fig. 4K-L/Fig. 4L.jpg]

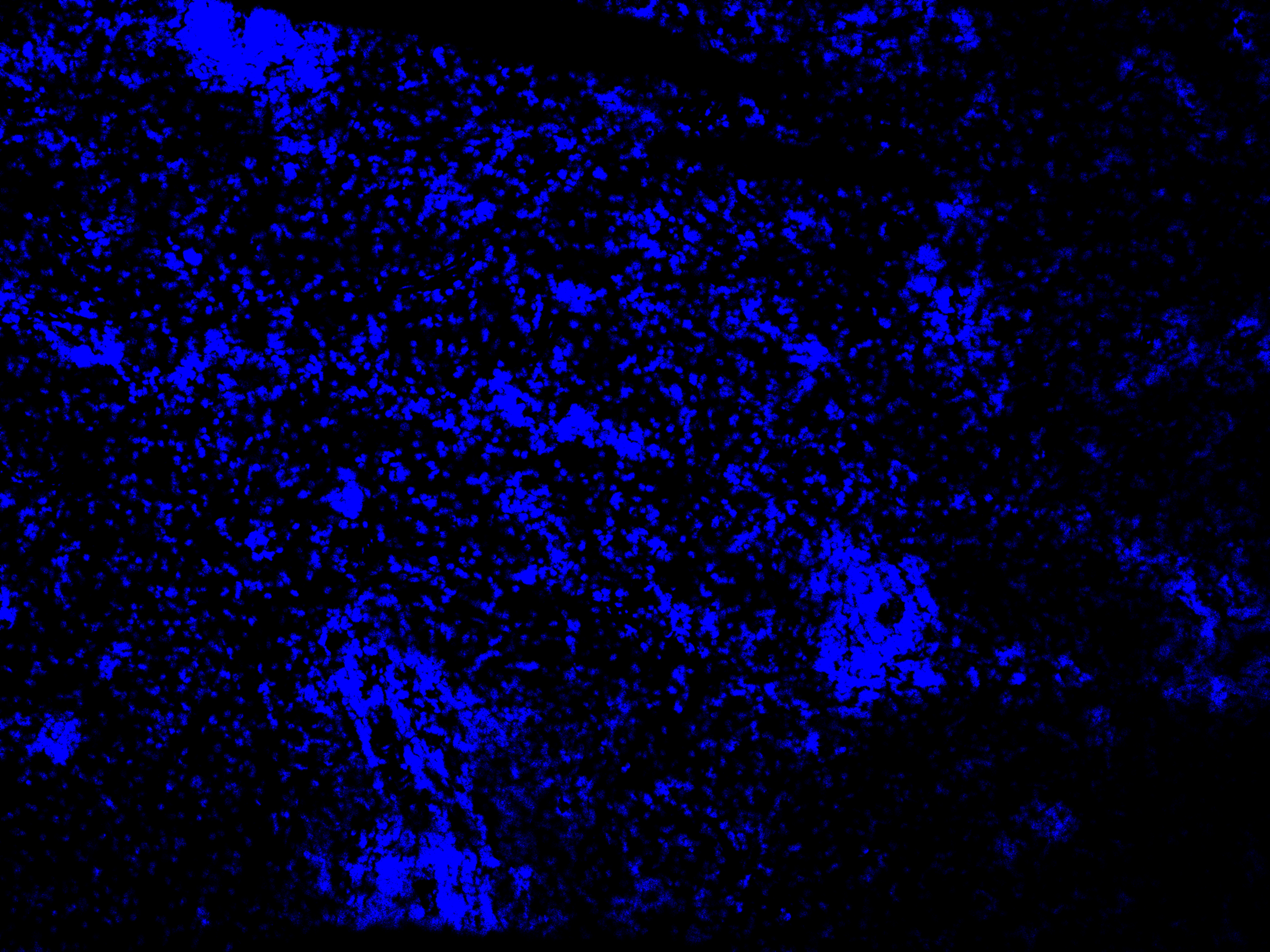

Supplement: Supplemental Information 13 — PKH67-labeled HF-MSCs were rarely seen in the spleen and kidney. [file peerj-10-12872-s013.zip › Fig. 4M-N/Fig. 4M.jpg]

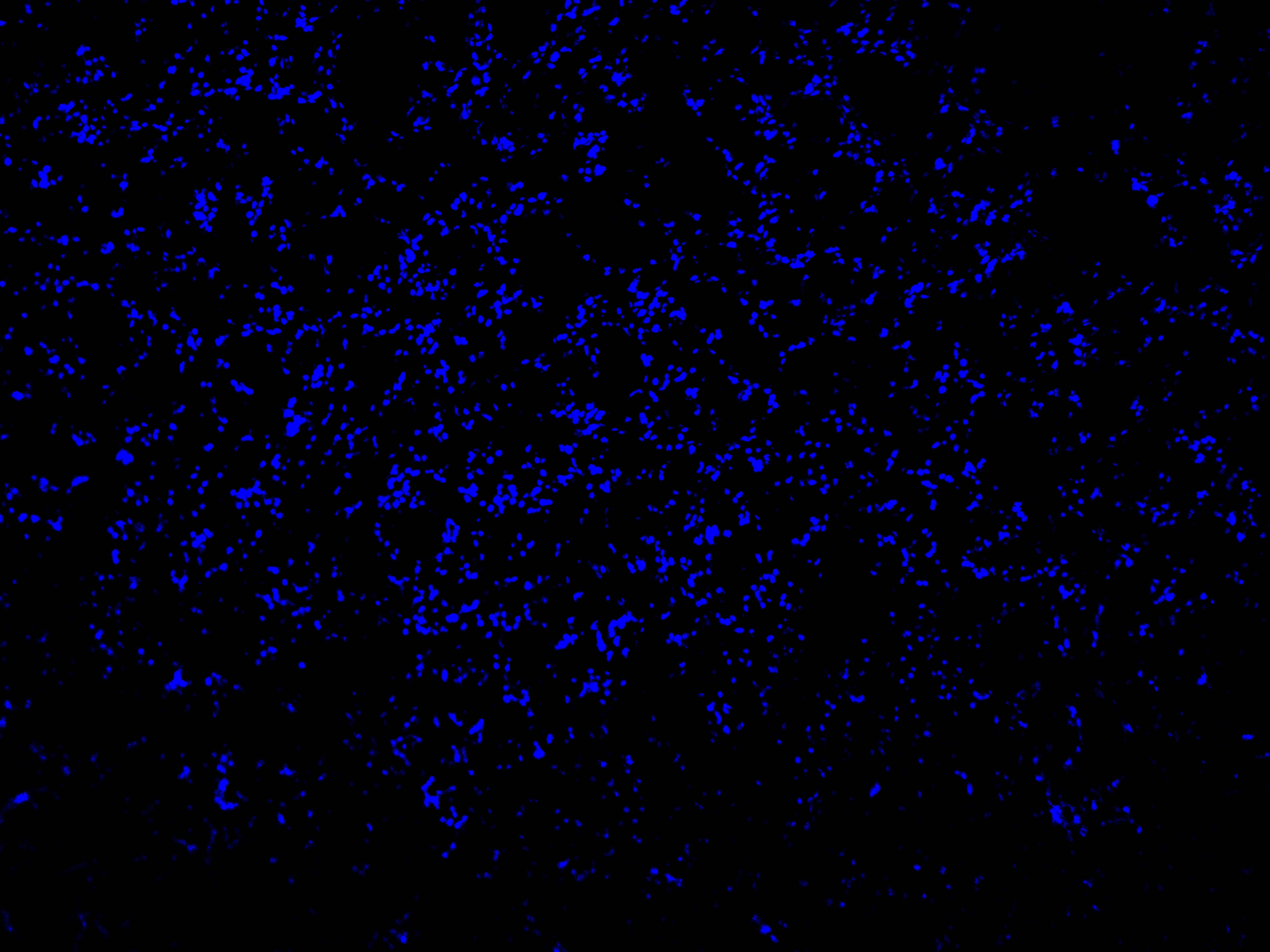

Supplement: Supplemental Information 13 — PKH67-labeled HF-MSCs were rarely seen in the spleen and kidney. [file peerj-10-12872-s013.zip › Fig. 4M-N/Fig. 4N.jpg]

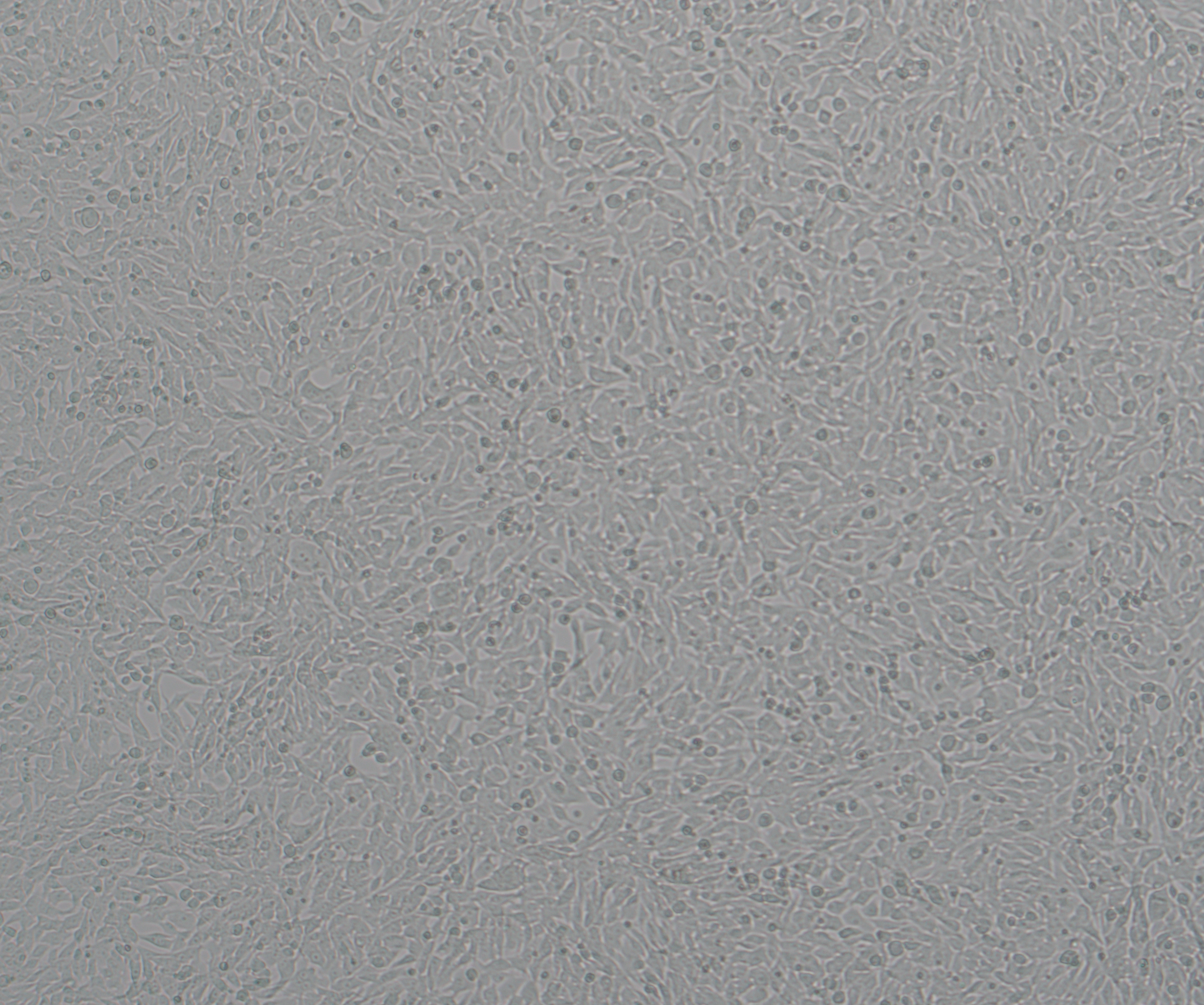

Supplement: Supplemental Information 14 — (A): Quiescent JS1 cells are in a static state, with irregular shapes, and the cell bodies are ovular or irregular; (B): The activated JS1 cells have enlarged cell bodies, protruding slender pseudopods, with a star-shaped appearance. [file peerj-10-12872-s014.zip › Fig. 5A-B/Fig. 5A.jpg]

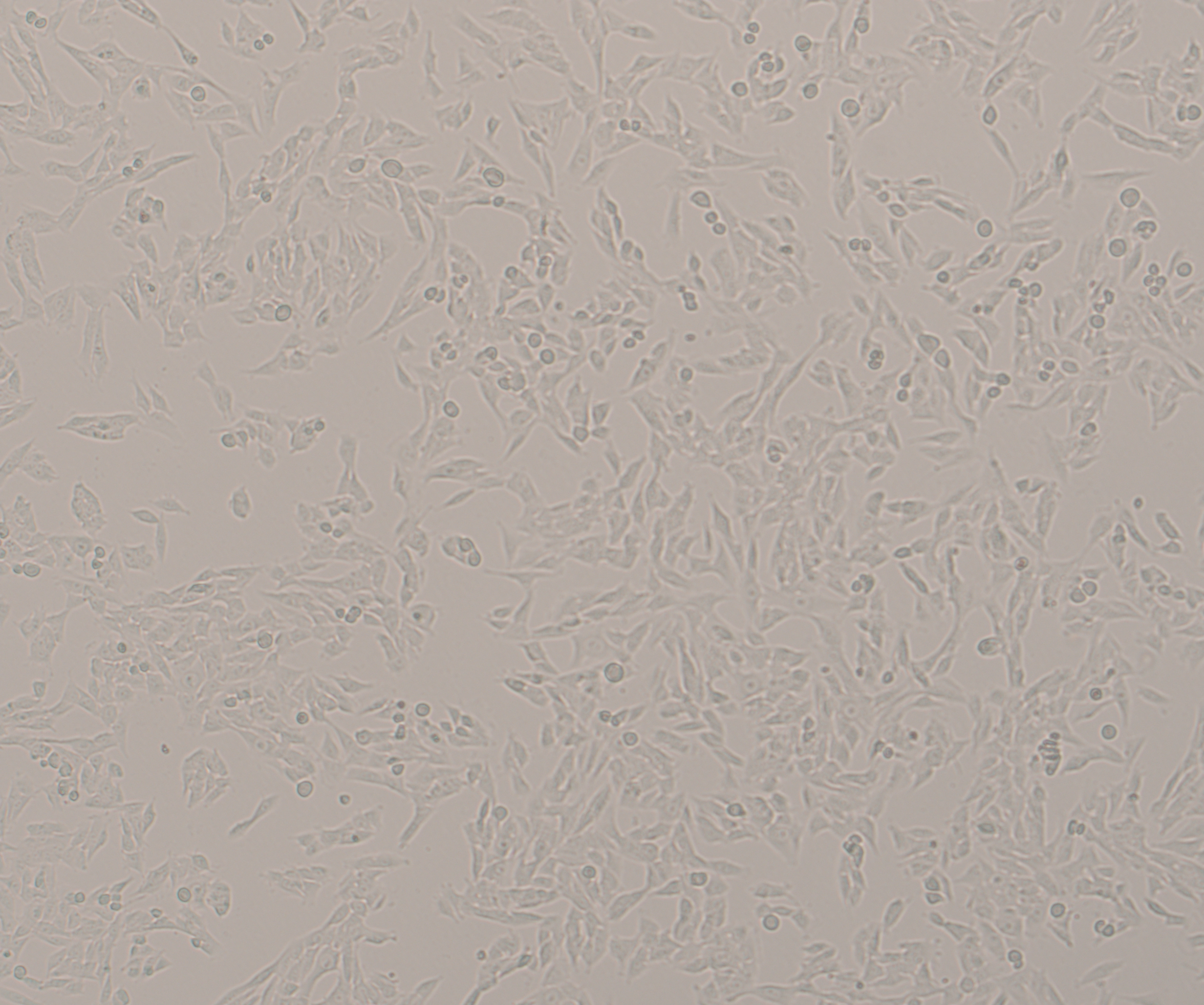

Supplement: Supplemental Information 14 — (A): Quiescent JS1 cells are in a static state, with irregular shapes, and the cell bodies are ovular or irregular; (B): The activated JS1 cells have enlarged cell bodies, protruding slender pseudopods, with a star-shaped appearance. [file peerj-10-12872-s014.zip › Fig. 5A-B/Fig. 5B.jpg]

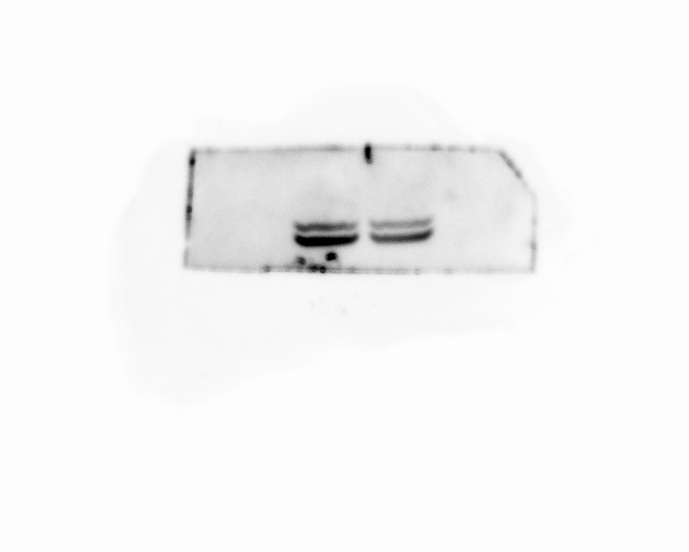

Supplement: Supplemental Information 15 — The protein expression of p-smad23 and TGF-β [file peerj-10-12872-s015.zip › Fig. 5C-p-smad23-TGF-a┬/Fig. 5C-p-smad23.jpg]

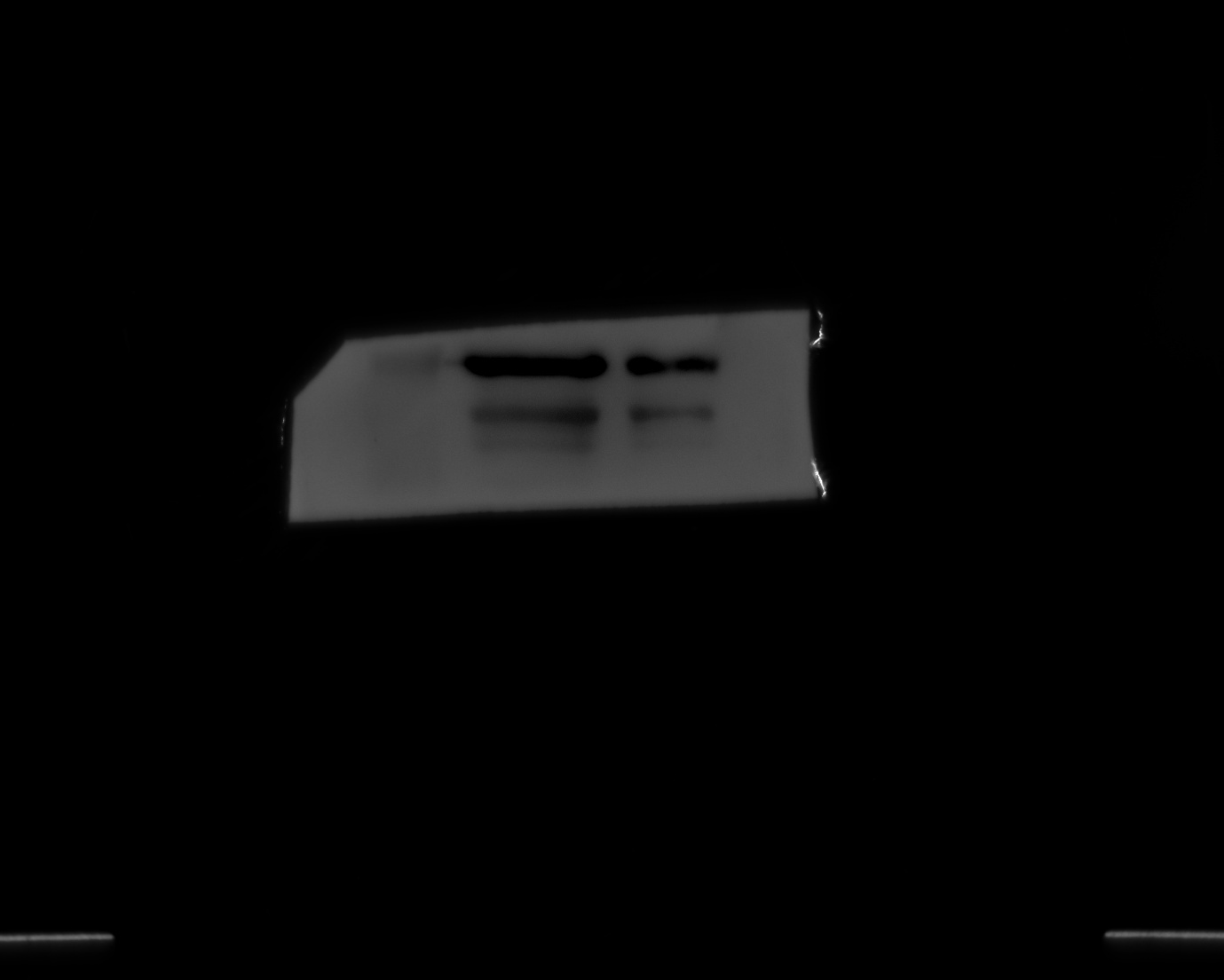

Supplement: Supplemental Information 15 — The protein expression of p-smad23 and TGF-β [file peerj-10-12872-s015.zip › Fig. 5C-p-smad23-TGF-a┬/Fig. 5C-TGF-a┬.jpg]

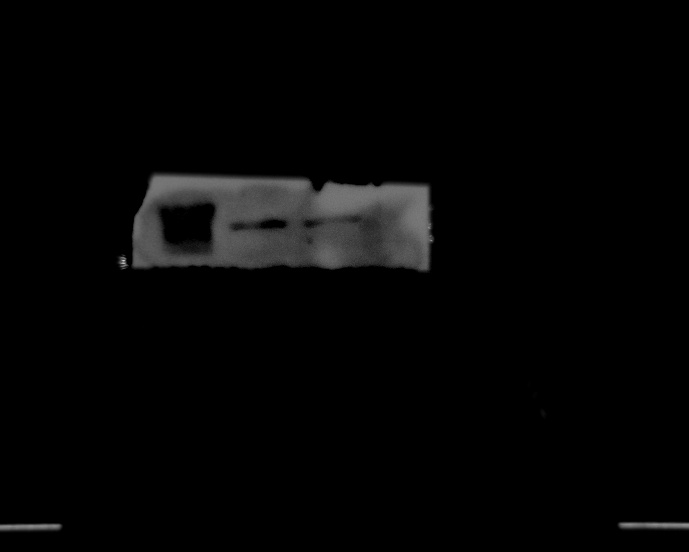

Supplement: Supplemental Information 16 — The protein expression of α-SMA and β-actin [file peerj-10-12872-s016.zip › Fig. 5C-a┴-SMA-a┬-actin/Fig. 5C-a┴-SMA.jpg]

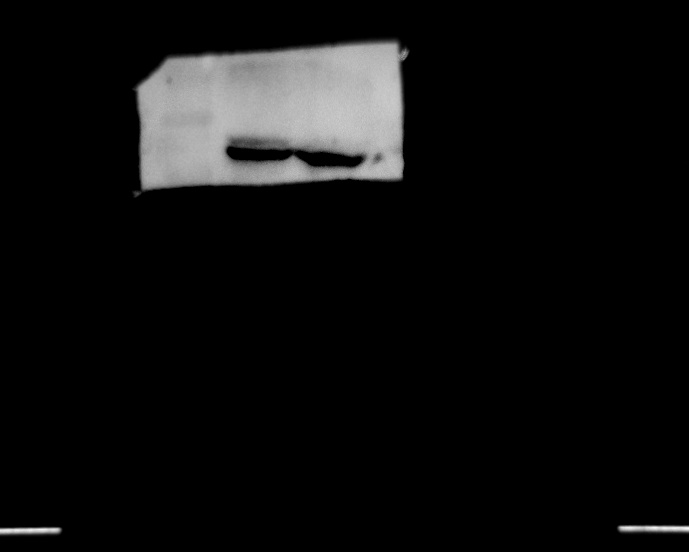

Supplement: Supplemental Information 16 — The protein expression of α-SMA and β-actin [file peerj-10-12872-s016.zip › Fig. 5C-a┴-SMA-a┬-actin/Fig. 5C-a┬-actin.jpg]

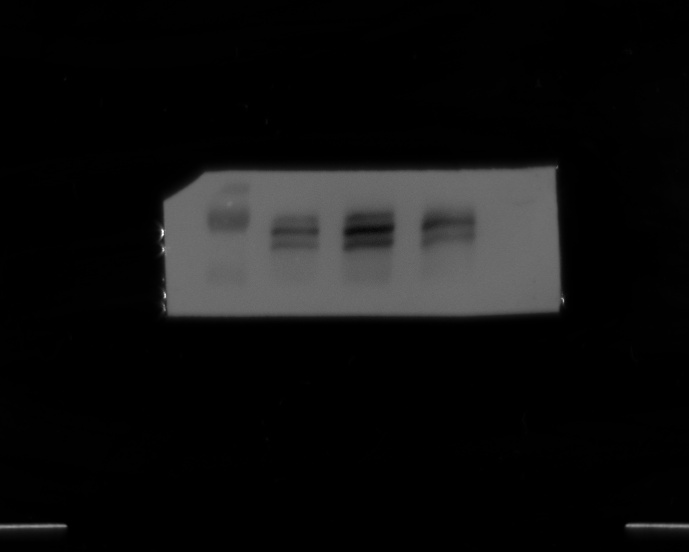

Supplement: Supplemental Information 18 — The protein expression of p-smad23 and TGF-β [file peerj-10-12872-s018.zip › Fig. 6A-p-smad23-TGF-a┬/Fig. 6A-p-smad23.jpg]

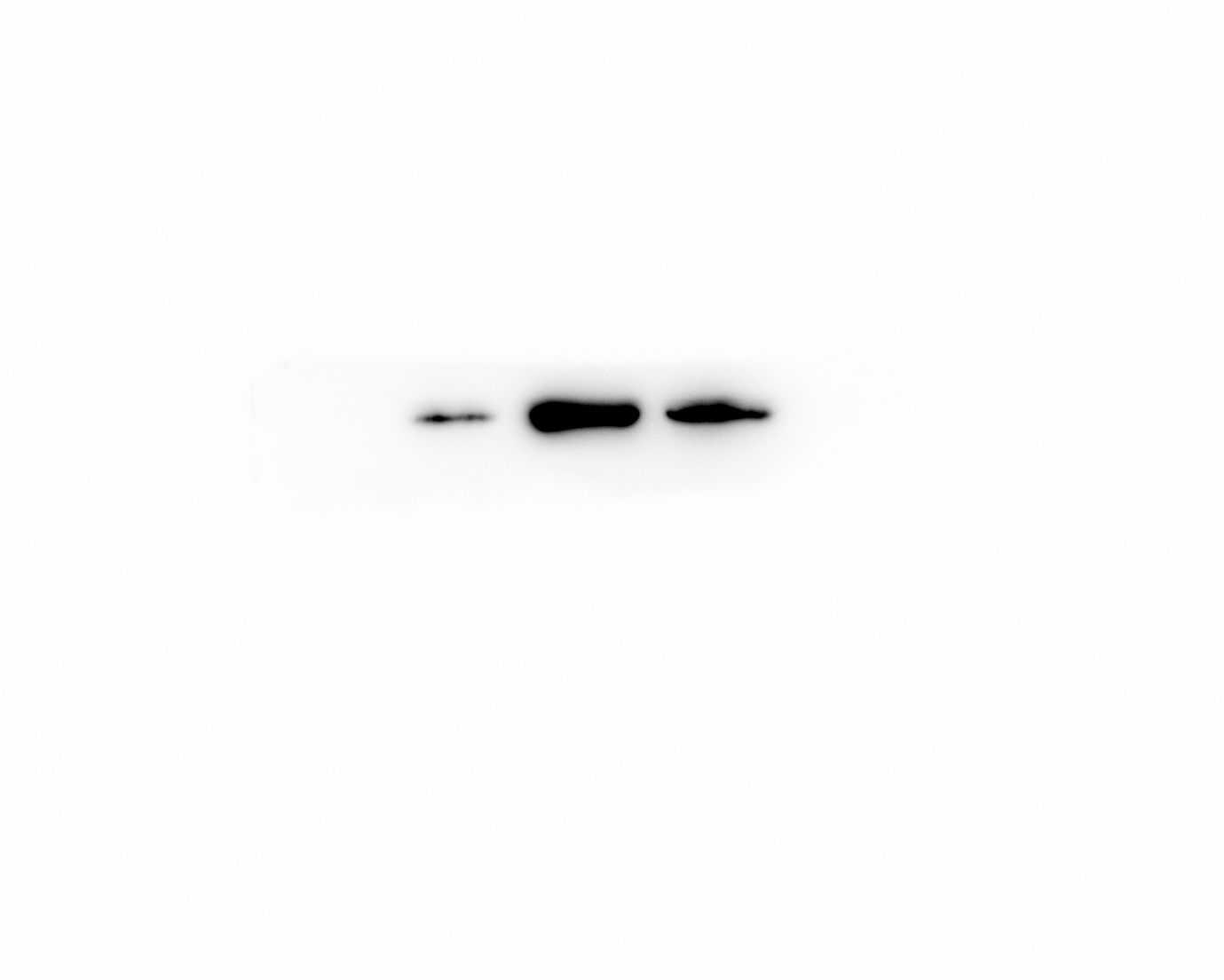

Supplement: Supplemental Information 18 — The protein expression of p-smad23 and TGF-β [file peerj-10-12872-s018.zip › Fig. 6A-p-smad23-TGF-a┬/Fig. 6A-TGF-a┬.jpg]

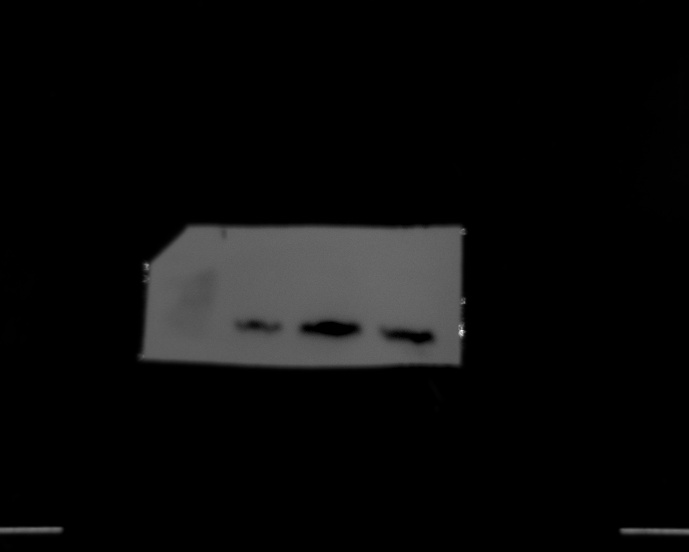

Supplement: Supplemental Information 19 — The protein expression of α-SMA and β-actin [file peerj-10-12872-s019.zip › Fig. 6A-a┴-SMA-a┬-actin/Fig. 6A-a┴-SMA.jpg]

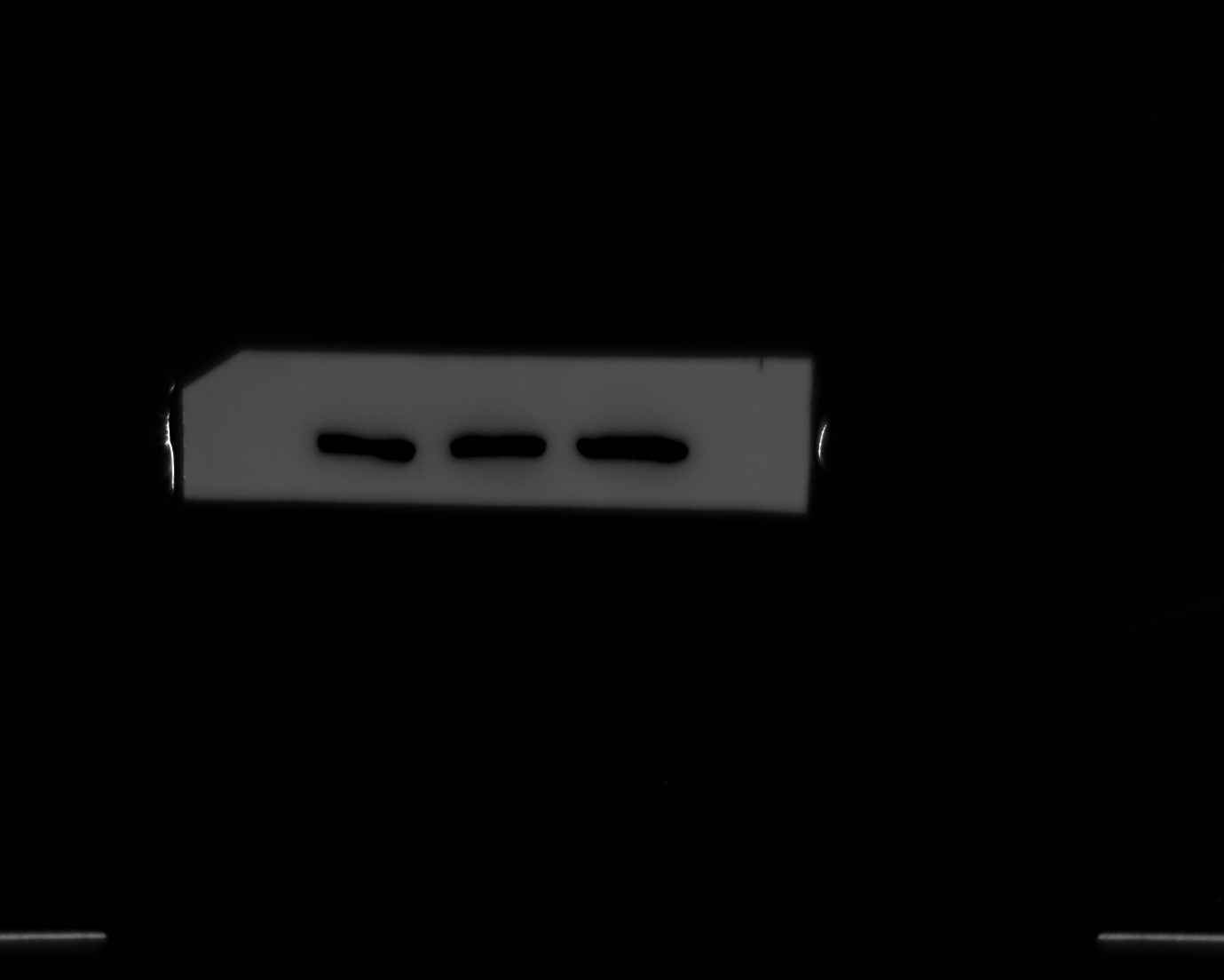

Supplement: Supplemental Information 19 — The protein expression of α-SMA and β-actin [file peerj-10-12872-s019.zip › Fig. 6A-a┴-SMA-a┬-actin/Fig. 6A-a┬-actin.jpg]
